# The inactive X chromosome is epigenetically unstable and transcriptionally labile in breast cancer

**DOI:** 10.1101/gr.185926.114

**Published:** 2015-04

**Authors:** Ronan Chaligné, Tatiana Popova, Marco-Antonio Mendoza-Parra, Mohamed-Ashick M. Saleem, David Gentien, Kristen Ban, Tristan Piolot, Olivier Leroy, Odette Mariani, Hinrich Gronemeyer, Anne Vincent-Salomon, Marc-Henri Stern, Edith Heard

**Affiliations:** 1 Centre de Recherche, Institut Curie, 75248 Paris Cedex 05, France;; 2 Centre National de la Recherche Scientifique, Unité Mixte de Recherche 3215, Institut Curie, 75248 Paris Cedex 05, France;; 3 Institut National de la Santé et de la Recherche Médicale U934, Institut Curie, 75248 Paris Cedex 05, France;; 4 Equipe Labellisée Ligue Contre le Cancer, UMR3215, 75248 Paris Cedex 05, France;; 5 Institut National de la Santé et de la Recherche Médicale U830, Institut Curie, 75248 Paris Cedex 05, France;; 6 Institut de Génétique et de Biologie Moléculaire et Cellulaire, Equipe Labellisée Ligue Contre le Cancer, Centre National de la Recherche Scientifique UMR 7104, Institut National de la Santé et de la Recherche Médicale U964, University of Strasbourg, 67404 Illkirch Cedex, France;; 7 Department of Tumor Biology, Institut Curie, 75248 Paris Cedex 05, France;; 8 Plate-forme d'Imagerie Cellulaire et Tissulaire at BDD (Pict@BDD), Institut Curie, 75248 Paris Cedex 05, France

## Abstract

Disappearance of the Barr body is considered a hallmark of cancer, although whether this corresponds to genetic loss or to epigenetic instability and transcriptional reactivation is unclear. Here we show that breast tumors and cell lines frequently display major epigenetic instability of the inactive X chromosome, with highly abnormal 3D nuclear organization and global perturbations of heterochromatin, including gain of euchromatic marks and aberrant distributions of repressive marks such as H3K27me3 and promoter DNA methylation. Genome-wide profiling of chromatin and transcription reveal modified epigenomic landscapes in cancer cells and a significant degree of aberrant gene activity from the inactive X chromosome, including several genes involved in cancer promotion. We demonstrate that many of these genes are aberrantly reactivated in primary breast tumors, and we further demonstrate that epigenetic instability of the inactive X can lead to perturbed dosage of X-linked factors. Taken together, our study provides the first integrated analysis of the inactive X chromosome in the context of breast cancer and establishes that epigenetic erosion of the inactive X can lead to the disappearance of the Barr body in breast cancer cells. This work offers new insights and opens up the possibility of exploiting the inactive X chromosome as an epigenetic biomarker at the molecular and cytological levels in cancer.

There is increasing evidence that epigenetic modifications, such as changes in DNA methylation, chromatin structure, noncoding RNAs, and nuclear organization, accompany tumorigenesis (De Carvalho et al. 2012; for review, see Shen and Laird 2013). Even tumors with relatively normal karyotypes can show dramatically perturbed nuclear structures (Huang et al. 1997; for review, see Zink et al. 2004). In theory, epigenetic changes could lead to inactivation of tumor suppressor genes, aberrant expression or function of oncogenes, or more global gene expression changes that perturb genome function, thereby contributing to cancer progression. However, despite the possible use of epigenetic changes as prognostic markers (Elsheikh et al. 2009) or even as therapeutic targets (e.g., Schenk et al. 2012; Zhang et al. 2012), the full extent of epigenetic changes in cancer remains poorly explored.

The inactive X chromosome (Xi), also known as the Barr body, provides an outstanding example of an epigenetic nuclear landmark that is disrupted in cancer. The disappearance of the Barr body in breast tumors was noted many decades ago (Barr and Moore 1957; Perry 1972; Smethurst et al. 1981). To date, only genetic instability had been clearly demonstrated as a cause for Barr body loss (Ganesan et al. 2002; Sirchia et al. 2005; Vincent-Salomon et al. 2007; Xiao et al. 2007; and for review, see Pageau et al. 2007). Past work had implicated *BRCA1*, a major hereditary factor predisposing to breast and ovarian cancer development and a key player in the maintenance of genome integrity (for review, see O'Donovan and Livingston 2010), in promoting *XIST* RNA coating of the Xi and its epigenetic stability (Ganesan et al. 2002; Silver et al. 2007). However, subsequent work in *BRCA1*-deficient tumors indicated that Barr body loss was usually due to genetic loss of the Xi and duplication of the Xa rather than to Xi reactivation and epigenetic instability (Sirchia et al. 2005; Vincent-Salomon et al. 2007; Xiao et al. 2007). *BRCA1*-deficient cancers are usually of the basal-like carcinoma (BLC) subtype, a high-grade, genetically unstable, invasive ductal carcinoma. Indeed, when the genetic status of the X chromosome was explored in BLCs (Richardson et al. 2006), genetic instability/loss of the Xi was found to be a frequent event in both sporadic and *BRCA1*^−/−^ associated BLCs. Luminal (A and B, expressing hormonal receptors) and HER2 (encoded by *ERBB2*) amplified molecular subtypes of invasive ductal carcinoma are more genetically stable and show less frequent loss of the inactive X chromosome (Perou et al. 2000; Turner and Reis-Filho 2006). However, little is known about the epigenetic status of the inactive X in breast cancers and the extent to which epigenetic instability might account for Barr body disappearance in some cases.

X-chromosome inactivation (XCI) ensures dosage compensation for X-linked gene products between XX females and XY males (Lyon 1961). It is a developmentally regulated process that depends on the action of a noncoding RNA, *Xist* (X-inactive-specific-transcript), which becomes up-regulated on one of the two X chromosomes, coating it in *cis* and inducing gene silencing. *Xist* RNA accumulation on the future inactive X rapidly creates a silent nuclear compartment that is depleted of RNA Polymerase II (RNA Pol II), transcription factors, and transcription (as detected by Cot-1 RNA). X-linked genes become repressed during the early stages of XCI (Chaumeil et al. 2006; Clemson et al. 2006; Chow et al. 2010). *Xist* RNA also induces a cascade of chromatin changes, involving Polycomb group proteins and other complexes, and results in various histone modifications, such as the hypoacetylation of histones 3 and 4, trimethylation of histone 3 lysine 27 (H3K27me3), and the loss of di- and trimethylation at histone 3 lysine 4 (H3K4me2/3) (Csankovszki et al. 1999; Heard et al. 2001; Boggs et al. 2002). Promoter DNA methylation of X-linked genes occurs downstream from *Xist* RNA coating, with gene-specific timing of promoter methylation (Gendrel et al. 2013). The Xi adopts a unique three-dimensional (3D) chromosome organization that is dependent on *Xist* RNA (Splinter et al. 2011; for review, see Chow and Heard 2010). Furthermore, the chromatin landscape of the inactive X has been investigated in adult human cells and seems to be divided into large blocks of H3K9me3 or H3K27me3 (Chadwick 2007; Chadwick and Willard 2004). In somatic cells, the majority of X-linked genes are stably repressed on the Xi, with spontaneous reactivation of single genes being observed at a frequency of <10^−8^, presumably due to synergistic epigenetic mechanisms (Csankovszki et al. 2001). However, a subset of genes can escape XCI in somatic cells (Carrel and Willard 2005; Kucera et al. 2011; Cotton et al. 2013). In cancer, aberrant escape from XCI has previously been speculated to occur (Pageau et al. 2007; Agrelo and Wutz 2010; Carone and Lawrence 2013; Yildirim et al. 2013). However, the extent to which the normally stable epigenetic state of the Xi is perturbed in cancer has never been systematically explored.

The X chromosome is of interest from a cancer perspective. First, several of the approximately 1000 genes located on the X have been implicated in cancer, including the cancer/testis (C/T) genes (Grigoriadis et al. 2009); tumor suppressors such as *AMER1* (also known as *WTX*), *FOXP3* (Bennett et al. 2001; Rivera et al. 2007); chromatin remodelers related to disease, e.g., *ATRX*; or chromatin modifying factors, e.g., *KDM6A* (also known as *UTX*)*, PHF8, HDAC8* (Nakagawa et al. 2007; for reviews, see Agrelo and Wutz 2010; Portela and Esteller 2010). A few of these genes are known to escape X inactivation in normal cells (e.g., *KDM6A*), but most are normally stably repressed on the inactive X. In the cases of *AMER1* and *FOXP3*, tumorigenesis has been linked to clonal expansion of cells in which the wild-type copy is on the inactive X in female patients heterozygous for a mutation (Bennett et al. 2001; Rivera et al. 2007).

Although reactivation of X-linked genes has been previously hypothesized to occur in a cancer context (Spatz et al. 2004), few actual examples have been reported, presumably due to the technical challenges in specifically detecting the Xi. For example, deletion of *Xist* was reported to lead to hematological dysplasia and leukemia in mice; however, the allele-specific transcriptional activity of the inactive X chromosome and its heterochromatin structure were not examined (Yildirim et al. 2013). In another study, reactivation of the X-linked *MPP1* gene and disrupted *XIST* expression were reported in an ovarian cancer cell line (Kawakami et al. 2004). In breast tumors, DNA hypomethylation and abnormal expression of a single X-linked gene analyzed, *VBP1,* was detected on the Xi (Richardson et al. 2006). A systematic analysis of the transcriptional and epigenetic status of the Xi in breast tumors has been lacking however. Here we perform an integrated analysis of gene expression, chromatin status, and nuclear organization of the inactive X chromosome in breast cancer, using allele-specific and single-cell approaches.

## Results

### Aberrant nuclear organization of the inactive X chromosome in breast cancer cells

**Figure 1.**
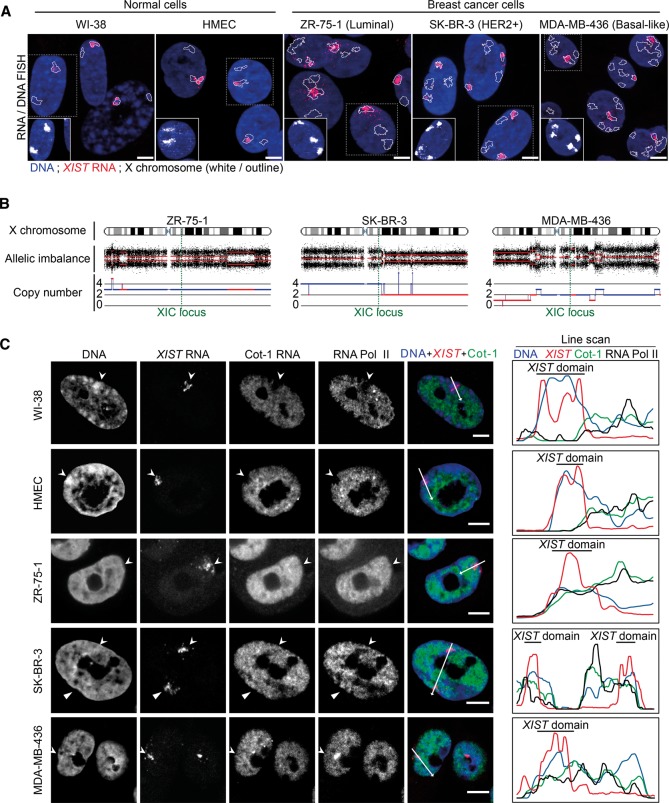
The *XIST*-coated X-chromosome silent compartment is severely disrupted in breast cancer cell lines. (*A*) Z-projections of sequential 3D RNA/DNA FISH show examples of *XIST* RNA coating (red) and X-chromosome territories (white or outlined) in normal (WI-38 and HMEC) and breast cancer cell lines (ZR-75-1, SK-BR-3, and MDA-MB-436). Scale bar, 5 µm. (*B*) Human SNP Array 6.0 (Affymetrix) genomic analysis (Popova et al. 2009) shows the copy number and allelic imbalance of X-chromosome fragments in breast cancer cell lines. The XIC locus is indicated with a green dotted line. (*C*) Immuno-RNA FISH using anti-RNA Pol II antibody, *XIST*/Cot-1 RNA FISH, and DAPI staining show the level of exclusion of RNA Pol II and Cot-1 RNA, as well as the level of chromatin compaction (i.e., Barr body) on *XIST* RNA domains (arrowheads) in normal and breast cancer cell lines. On the *right*, line scans (white arrows) show the relative levels of Cot-1 RNA (green), RNA Pol II (black), and DNA density (blue) at the *XIST* domain (black bar). Scale bar, 5 µm.

To evaluate the status of the inactive X chromosome in different types of breast cancer, we selected three cell lines that represent the main breast cancer molecular subtypes: ZR-75-1 (luminal), SK-BR-3 (HER2+), and MDA-MB-436 (Basal-Like Carcinoma [BLC], *BRCA1* null). WI-38 (embryonic lung fibroblasts) and Human Mammary Epithelial Cells (HMECs) were analyzed in parallel as nonmalignant (“normal”) female primary cells. Using RNA FISH, we found that ZR-75-1 and MDA-MB-436 cell lines possess one *XIST* RNA domain, whereas SK-BR-3 cells have two domains. X-chromosome paint DNA FISH combined with *XIST* RNA FISH, and 3D microscopy revealed that *XIST* RNA signals overlapped to a great extent with the X chromosome DNA in both normal and tumor cell lines. However, punctate *XIST* RNA signals beyond the X-chromosome territory could be detected in the tumor cell lines, particularly in ZR-75-1 and MDA-MB-436 (Fig. 1A; Supplemental Fig. S1A). RT-qPCR revealed that *XIST* was expressed at slightly lower levels in the tumor cell lines, and the associated RNA FISH signal was slightly weaker and was more dispersed in the breast cancer cell lines (Supplemental Fig. S1B,C,E). Importantly, all of the tumor cell lines revealed a markedly weaker DNA enrichment of the Barr body (Supplemental Fig. S1D,E).

Given the complex genomes of breast cancer cells, we investigated the precise genetic constitution of the active and inactive X chromosomes using single nucleotide polymorphism array (Human SNP Array 6.0) analysis and DNA FISH (Fig. 1B; Supplemental Fig. S1F). ZR-75-1 contains three X-chromosome segments, each carrying an XIC/*XIST* locus, but *XIST* RNA coated only one of them, suggesting the presence of two Xa chromosomes and one Xi (in agreement with allelic imbalance of the XIC locus). SK-BR-3 possesses four X-chromosome fragments, each with an XIC locus, but only two are associated with *XIST* RNA. MDA-MB-436 displayed the most complex situation, with six X-chromosome fragments visible by DNA FISH on metaphase spreads, but with only two XIC loci and one *XIST* RNA domain (Fig. 1A,B; Supplemental Fig. S1F). We also evaluated X-chromosome constitution in these cell lines through the expression of two X-linked genes: *KDM5C*, known to escape from XCI, and *HUWE1*, subject to XCI (Cotton et al. 2013). Our observations concur with the expected expression profiles in the two normal and three cancer cell lines, i.e., *KDM5C* is expressed from all X chromosome fragments that carried the gene, and *HUWE1* is expressed only from the non-*XIST* RNA-coated X fragments that carried it (Supplemental Fig. S1G). Thus, all three tumor cell lines contain at least one fragment of an Xi chromosome.

We then investigated whether *XIST* RNA-coated Xi fragments were depleted for RNA Pol II and Cot-1 RNA as previously described for the Xi in female somatic cells (Chaumeil et al. 2006; Clemson et al. 2006; Chow et al. 2010). In WI-38 and HMEC cells, both Cot-1 RNA and RNA Pol II were excluded from the *XIST* domain, which was associated with a DAPI-dense, heterochromatic Barr body. However, all tumor cells showed a frequent absence of a DAPI-dense Barr body and a defective depletion of Cot-1 RNA and RNA Pol II within the *XIST* domain (Fig. 1C; Supplemental Figs. S1H–K, S2A,C). Together, these results reveal major aberrations of nuclear organization and chromosome condensation of the *XIST* RNA-coated X chromosome in breast cancer cells.

### Aberrant chromatin hallmarks of the inactive X chromosome in breast cancer cell lines

**Figure 2.**
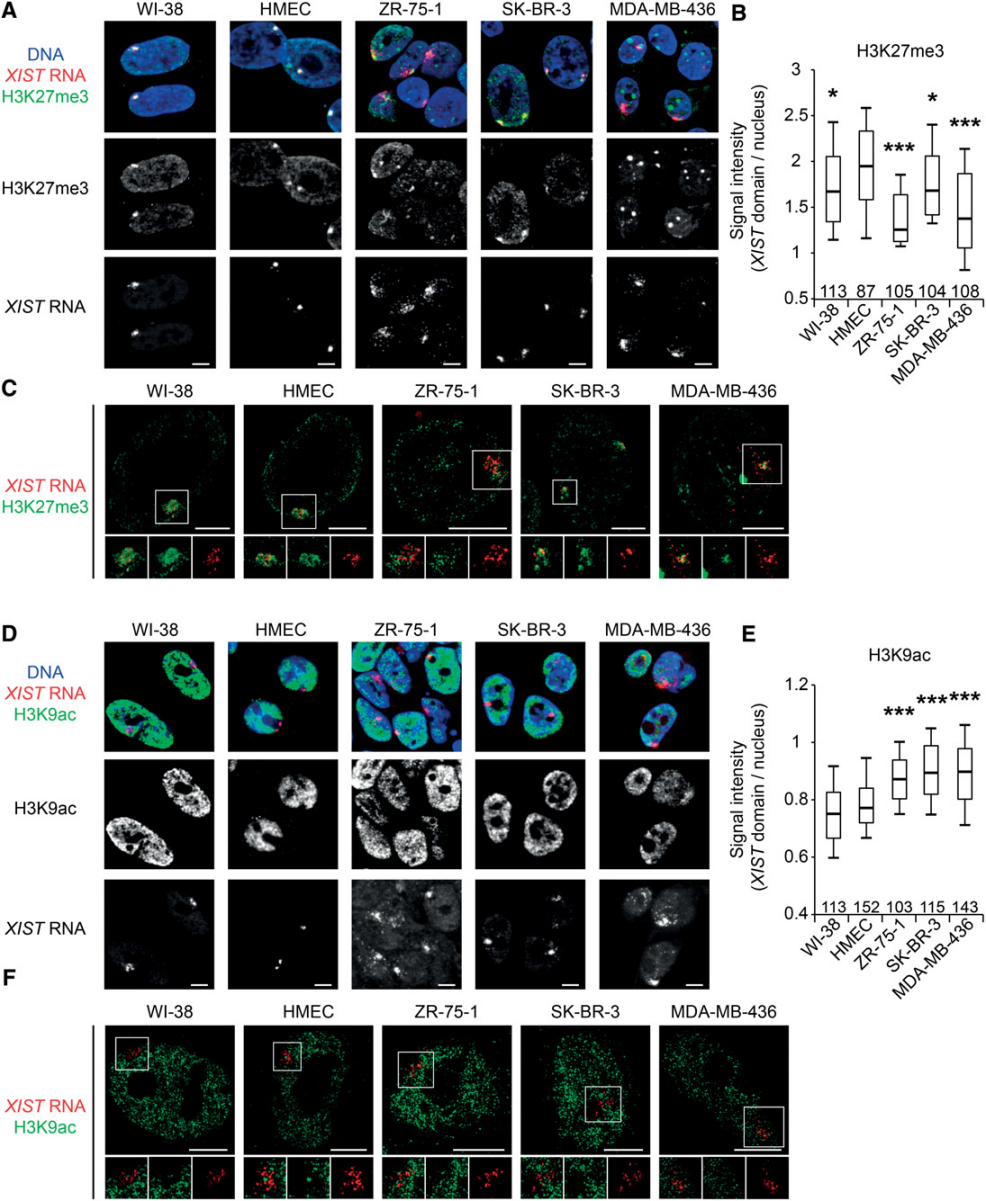
H3K27me3 and H3K9ac profiles associated with *XIST*-coated X chromosomes are impaired in breast cancer cell lines. (*A*) Z-projections of 3D immuno-RNA FISH show representative examples of the level of H3K27me3 enrichment (green) on *XIST* RNA domains (red) in normal (WI-38 and HMEC) and breast cancer cell lines (ZR-75-1, SK-BR-3, and MDA-MB-436). NB: In MDA-MB-436, the highly H3K27me3 enriched bodies visible in each nucleus do not belong to the X chromosome (nor in metaphase [Fig. 3C] or in interphase [Supplemental Fig. S3F]). (*B*) Boxplot shows the levels of H3K27me3 enrichment on *XIST* domains relative to the rest of the nucleus. Numbers of analyzed nuclei are shown *above* the *x*-axis. For details on quantification method see Supplemental Figure S2A,B. (*C*) High-resolution immuno-RNA FISH shows representative examples of H3K27me3 enrichment (green) on *XIST* RNA domains (red) in normal and breast cancer cell lines. *Insets* for H3K27me3, *XIST* RNA, and merge are shown *below* each cell line. (*D*) Single section of 3D immuno-RNA FISH shows representative examples of the level of H3K9ac depletion (green) on *XIST* RNA domains (red) in normal and breast cancer cell lines. (*E*) Boxplot shows the levels of H3K9ac depletion on *XIST* domains relative to the rest of the nucleus. The numbers of analyzed nuclei are shown *above* the *x*-axis. For details on the quantification method, see Supplemental Figure S2A,C. (*F*) High-resolution immuno-RNA FISH shows representative examples of H3K9ac depletion (green) on *XIST* RNA domains (red) in normal and breast cancer cell lines. *Insets* for H3K9ac, *XIST* RNA, and merge are shown *below* each cell line. (Boxplots) Upper whisker represents 90%, upper quartile 75%, median 50%, lower quartile 25%, and lower whisker 10% of the data set for each cell line. (***) *P* < 0.001; (**) *P* < 0.01; (*) *P* < 0.05 using the Student's *t*-test. All data sets are compared with HMEC data set. Scale bar, 5 µm.

We next investigated whether heterochromatic hallmarks of the Xi were preserved. Detection of H3K27me3 by IF combined with *XIST* RNA FISH revealed a marked lack of H3K27me3 enrichment at the *XIST*-coated chromosome in all three tumor cell lines (Fig. 2A). In HMECs, H3K27me3 is about twofold more enriched on the Xi than on the non-*XIST*-coated genome (Fig. 2B; Supplemental Fig. S2A,B). In tumor cells, the lowest enrichment was found in ZR-75-1 and MDA-MB-436 with a median of 1.25 and 1.37-fold, respectively, whereas for SK-BR-3 it is 1.68 (Fig. 2A,B; Supplemental Fig. S2I). Decreased H3K27me3 enrichment at the *XIST* domain was further supported by super resolution structured illumination microscopy (SIM) (Fig. 2C). Indeed, ZR-75-1 and MDA-MB-436 showed the lowest degree of *XIST* and H3K27me3 colocalization with a Pearson colocalization coefficient of 0.15, whereas SK-BR-3 had a coefficient at 0.35. HMEC and WI-38 displayed colocalization coefficients of 0.44 and 0.45, respectively (Supplemental Fig. S2D).

Depletion of euchromatic histone modifications is another hallmark of the Xi. Using IF combined with *XIST* RNA FISH, we found that H3K9 and H4 acetylation were present within the *XIST* RNA domain in tumor cells in contrast to normal cells (Fig. 2D,E; Supplemental Fig. S2E,F,I). The H3K4me2 mark was less perturbed, being globally absent from the Xi, except in ZR-75-1 cells (Supplemental Fig. S2G–I). Similar results were obtained for H3K4me3 staining (with, for example, median at 0.69 and 0.71, respectively, for HMEC and MDA-MB-436) (data not shown). Closer examination by SIM revealed that H3K9ac and *XIST* RNA signals were intermingled in the majority of breast cancer nuclei (Fig. 2F), whereas H3K4me2 was largely but not completely depleted within the *XIST* RNA compartment (Supplemental Fig. S2J). SIM of RNA Pol II also revealed substantial intermingled overlap with *XIST* RNA domains (Supplemental Fig. S2K). Thus, there is a major disruption of chromatin hallmarks over the *XIST* RNA-coated chromosome, most strikingly in ZR-75-1 and MDA-MB-436 cell lines. We confirmed that *XIST* is always expressed from only one allele, excluding the possibility of aberrant *XIST* expression and coating of the Xa instead of the Xi (Supplemental Fig. S3A). In summary, the heterochromatic structure of the Xi is disrupted in the three tumor cell lines to variable extents. The variability in Xi perturbation between cells was not found to be linked to a specific stage of the cell cycle (Supplemental Fig. S3B–D). Furthermore the global levels of histone modifications in the different cell lines did not correlate with the aberrant chromatin status of the Xi (Supplemental Fig. S3E).

**Figure 3.**
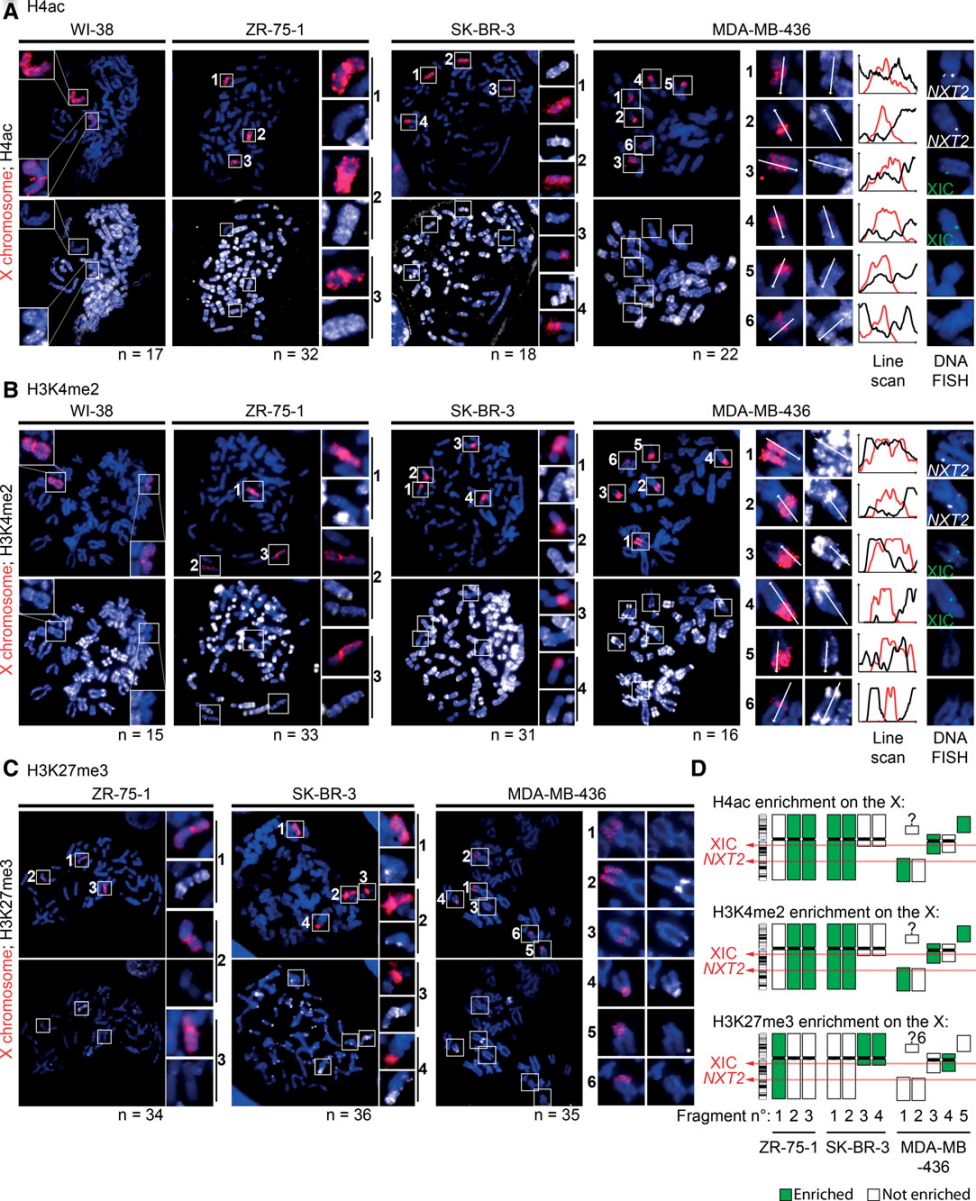
The inactive X chromosome is still epigenetically distinguishable from its active counterpart. (*A*) Representative examples of immunofluorescence show the status of H4ac (white) depletion/enrichment on X chromosomes (X-paint DNA FISH, red) on metaphase spreads from normal (WI-38) and breast cancer cell lines (ZR-75-1, SK-BR-3, and MDA-MB-436). On the *right*, MDA-MB-436 cells carry six X-chromosome fragments with a “2-by-2” homology, as assessed by the presence or absence of the *NXT2* (white) or XIC loci (green), and line scans show H4ac enrichment variation between these X-fragments and the neighboring autosomal regions. As expected, one X chromosome (Xi) lacks H4ac staining in normal WI-38 cells (and HMEC, not shown). ZR-75-1 and SK-BR-3 cell lines harbor a reduced H4ac staining on one and two X chromosomes, respectively, in agreement with the number of *XIST*-coated X chromosomes shown in Figure 1A. In MDA-MB-436 cells, homologous X-chromosome fragments (two containing the XIC locus, two containing the *NXT2* locus, and two with none of them) display opposite H4ac staining, suggesting that there is still one inactive and one active X chromosome linked to those loci, although fragmented. (*B*) Representative examples of immunofluorescence show the status of H3K4me2 (white) depletion/enrichment on X chromosomes (X-paint DNA FISH, red) on metaphase spreads from normal and breast cancer cell lines. On the *right*, line scans show H3K4me2 enrichment variation between the six X-fragments (for details, see *A*) and the neighboring autosomal regions in MDA-MB-436 cells. In each tumoral cell line, H3K4me2 depletion patterns follow the H4ac profiles found in *A*. (*C*) Representative examples of immunofluorescence show the status of H3K27me3 (white) enrichment on X chromosomes (X-paint DNA FISH, red) in metaphase spreads from breast cancer cell lines. ZR-75-1 and SK-BR-3 cell lines harbor an accumulation of H3K27me3 on one and two X chromosomes, respectively, in agreement with the number of *XIST*-coated X chromosomes shown in *A*. In MDA-MB-436 cells, H3K27me3 staining was only enriched on the X-chromosome fragment, where the XIC region lies. Indeed, RNA/DNA FISH analysis showed that this X fragment corresponds to the one coated by *XIST* RNA in interphase cells, which is not the case for the other fragments (Supplemental Fig. S3F). In SK-BR-3 and MDA-MB-436 cell lines, H3K27me3 spreads into the autosomal fragments translocated to the XIC-containing fragment. (*D*) Schematic view of H4ac, H3K4me2, and H3K27me3 patterns on X-chromosomes in the three tumor cell lines.

To specifically compare the chromatin status of the Xi and Xa in the tumor cell lines, we used metaphase spreads to monitor chromatin marks by IF followed by X-chromosome paint DNA FISH as described (Fig. 3; Keohane et al. 1996; Chaumeil et al. 2002). In all tumor cell lines, we could readily distinguish Xa from Xi fragments using H3K27me3, H4ac, and H3K4me2 (Fig. 3A–C). The only exception was MDA-MB-436, where from the two main Xi fragments, only the XIC-linked (and *XIST*-coated) fragment is enriched for H3K27me3 (Fig. 3C,D; Supplemental Fig. S3F), whereas the other (non-XIC-linked) X fragment lacked H3K27me3 enrichment, although it was still depleted for H4ac and H3K4me2. Thus, the XIC is required for H3K27me3 enrichment but is dispensable for depletion of euchromatin marks on the Xi in these cancer cells (Fig. 3A,B). We also noted from the analysis of metaphase spreads that in MDA-MB-436 and SK-BR-3 cells, where the Xi is translocated to an autosomal region, H3K27me3 enrichment was seen beyond the X chromosome paint signal, implying that it can spread aberrantly into autosomal regions (Fig. 3C).

### Reactivation of X-linked genes on the inactive X chromosome in breast cancer cell lines

**Figure 4.**
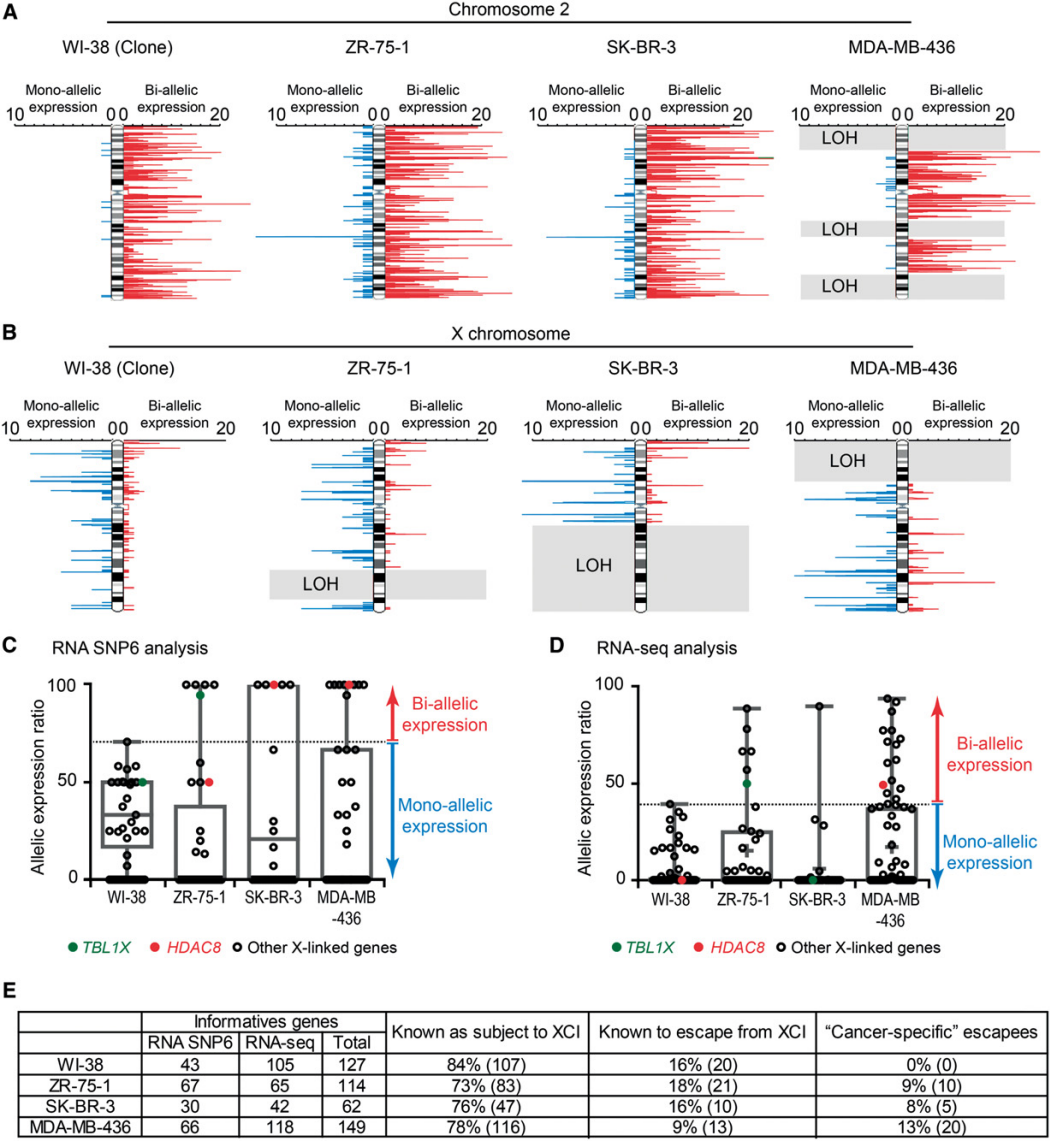
Abnormal reactivation of the inactive X chromosome in breast cancer cell lines. (*A*,*B*) RNA SNP6 analysis shows the expression status of an autosomal chromosome, as example Chromosome 2 (*A*), and the X chromosome (*B*) in normal (WI-38) and breast cancer cell lines (ZR-75-1, SK-BR-3, and MDA-MB-436). Red bars indicate biallelic expression, and blue bars indicate monoallelic expression. The bar length represents the number of expressed informative SNPs on a 50-SNP sliding window. Gray rectangles correspond to noninformative regions due to loss of heterozygosity (LOH). Two WI-38 subclones (#1 and #28), carrying an inactive X chromosome of opposite parental origin, show clear monoallelic expression from either the maternal or paternal X chromosome confirming the clonality of the subclones (see Supplemental Fig. S4B). Allele-specific PCR analysis also confirmed the clonality of the three breast tumor cell lines (see Supplemental Fig. S4C–E). (*C*) RNA SNP6 analysis shows levels of X-linked gene allelic expression. X-linked genes known as subject to XCI (Carrel and Willard 2005; Cotton et al. 2013) and/or considered as monoallelically expressed in WI-38 clones (i.e., for each informative gene, <2/3 of the SNPs were observed as biallelically expressed) are shown on the boxplots. (*D*) RNA-seq analysis shows levels of X-linked gene allelic expression. X-linked shown on the boxplots are known to be subject to XCI (Carrel and Willard 2005; Cotton et al. 2013) and/or are considered as monoallelically expressed in WI-38 clones (i.e., for each informative gene, the allelic expression ratio is <40, i.e., expressed <20% on one of the two alleles). (*E*) Summary of the informative genes identified by the RNA SNP6 and RNA-seq approaches. Genes “known as subject to XCI” or “known to escape from XCI” refer to previous studies (Carrel and Willard 2005; Cotton et al. 2013). WI-38 data correspond to the two clones.

We next assessed whether the heterochromatic disruption of the Xi observed in breast tumor cell lines corresponded to aberrant abnormal transcriptional activity from the Xi. To take advantage of SNPs that lie within introns of genes, we used an allele-specific transcriptional analysis based on nascent RNA hybridization to Human SNP Array 6.0 (henceforth called RNA SNP6) (Fig. 4A,B; Supplemental Fig. S4A; Gimelbrant et al. 2007). Due to the randomness of the XCI, clonal populations of cells are required to investigate Xi status. This was the case for all three tumor cell lines and for subclones derived from primary WI-38 cells (Supplemental Fig. S4B–E). In both WI-38 clones and the tumor cell lines, we saw the expected overall biallelic expression from autosomal regions (Chromosome 2 is shown as an example in Fig. 4A; Supplemental Fig. S4F). On the other hand, the X chromosome showed a globally monoallelic expression pattern in WI-38 clones, with the exception of genes in the pseudoautosomal regions that are known to behave as autosomes and to escape fully from XCI (Fig. 4B; Supplemental Fig. S4G). In tumor cells, we observed a generally monoallelic expression pattern from the X chromosome, although several regions showed biallelic expression, particularly in MDA-MB-436 cells (Fig. 4B). A gene-based analysis detected several previously described X-linked escapees (including *DHRSX, TRAPPC2*, *CD99*, or *KDM6A*) (Carrel and Willard 2005; Kucera et al. 2011; Cotton et al. 2013), confirming the efficiency of this approach. We used known escapees and genes subject to XCI (Carrel and Willard 2005; Cotton et al. 2013) to define a threshold to consider that a given X-linked gene is expressed from inactive and active alleles. Thus, we defined “cancer-specific” escapees as genes reactivated in at least one of the three cancer cell lines, but strictly expressed from the Xa in WI-38 clones and/or identified previously as subject to XCI (Fig. 4C). With these stringent criteria, we identified five, five, and nine “cancer-specific” escapees in the ZR-75-1, SK-BR-3, and MDA-MB-436 cells, respectively. To increase the number of informative X-linked genes evaluated, we also performed an RNA-seq analysis on mRNA from two additional WI-38 clones and the three tumor cell lines. We identified six, one, and 15 “cancer-specific” escapees in the ZR-75-1, SK-BR-3, and MDA-MB-436 lines, respectively (Fig. 4D). We validated Xi-linked reactivation for several of these genes (Supplemental Figs. S4H–J, S5A). In conclusion, although RNA SNP6 and RNA-seq analyses do not necessarily reveal exactly the same “cancer-specific” escapees (15%–23% overlap was found, depending on cell line) due to the different SNPs assessed by the two methods (mainly intronic and mainly exonic, respectively), the combination of both techniques allowed us to identify 10 (9% of informative X-linked genes), five (8%), and 20 (13%) Xi-linked genes as being abnormally reactivated in ZR-75-1, SK-BR-3, and MDA-MB-436 cell lines, respectively (Fig. 4E; Supplemental Table S1).

The preceding allele-specific analysis could not identify genes that are fully silenced in somatic cells and reactivated from only one allele in cancer cells, such as members of the C/T antigen family that show aberrant expression in cancer cells (Grigoriadis et al. 2009). By assessing the overall expression of C/T members, we found increased expression of several C/T antigens in the cancer cell lines but not in normal cells (Supplemental Fig. S5B). For one C/T antigen gene (*MAGEA6*), we used RNA FISH to show that this aberrant expression usually originated from the active rather than the inactive X in tumor cells (Supplemental Fig. S5C).

**Figure 5.**
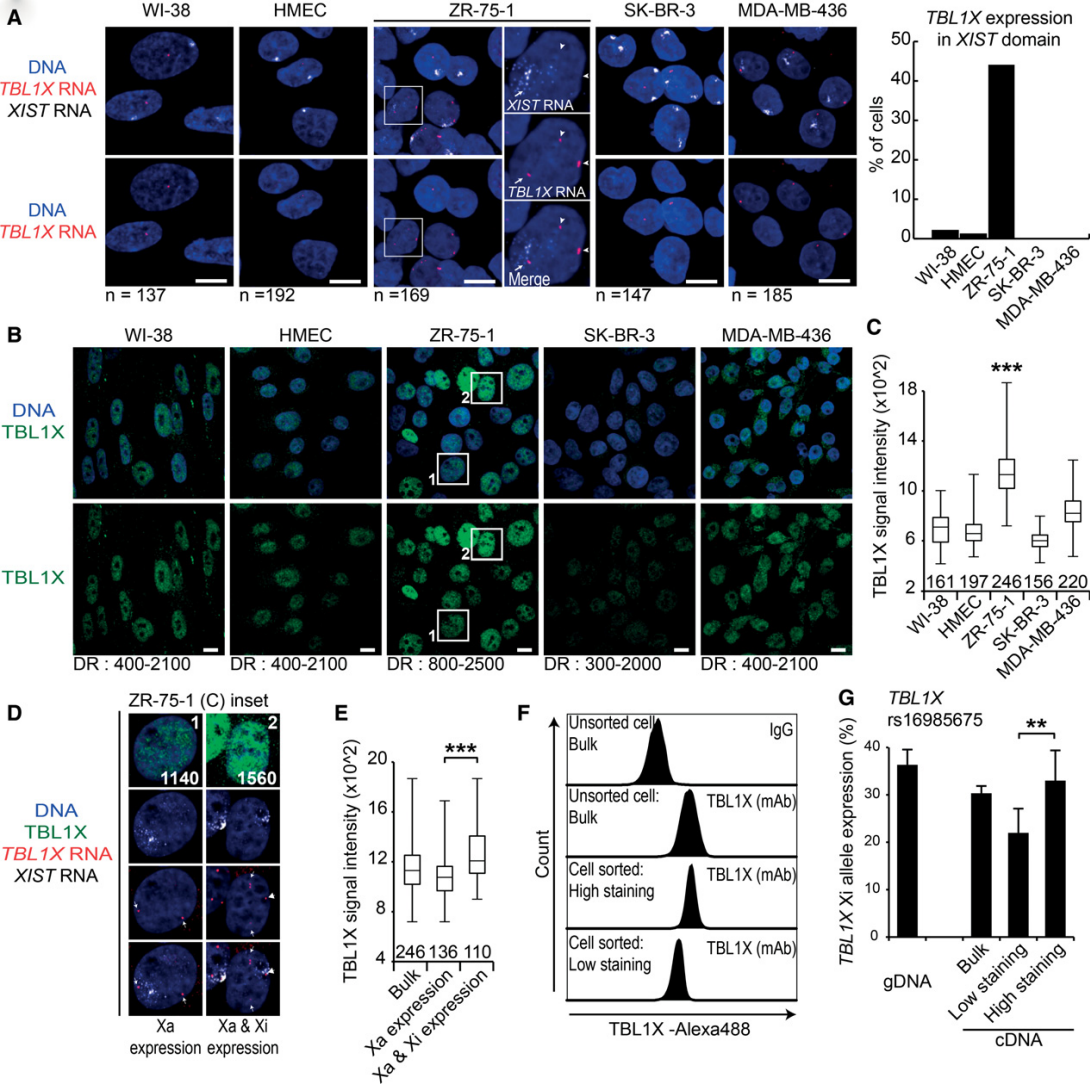
Reactivation of X-linked genes in breast cancer cell lines can lead to an increase of protein amount. (*A*) Z-projections of 3D RNA FISH show representative examples of *TBL1X* expression (red) at *XIST* domains (white) in normal (WI-38 and HMEC) and breast cancer cell lines (ZR-75-1, SK-BR-3, and MDA-MB-436). In ZR-75-1 cells, arrowheads indicate active X chromosomes and the arrow the *XIST*-coated chromosome. On the *right*, bar graph shows levels of *TBL1X* expression from *XIST* domains, with reactivation in ZR-75-1 cells. (*B*) Immunostaining shows TBL1X protein (green). The dynamic range (DR) of the brightness and contrast of each image (ImageJ) is indicated *below*. (*C*) Boxplot shows the intensity of TBL1X immunostaining for each cell line. The upper whisker represents the maximum value, upper quartile 75%, median 50%, lower quartile 25%, and lower whisker the minimum value of the data set. The number of nuclei analyzed is indicated *above* the *x*-axis. (***) *P* < 0.001 using the Student's *t*-test. WI-38, ZR-75-1, SK-BR-3, and MDA-MB-436 are compared with HMEC. (*D*) The *inset* of two ZR-75-1 nuclei from *C* shows a combination of TBL1X protein immunofluorescence staining (green) and RNA FISH for *TBL1X* (red) and *XIST* (gray). In the *left* nucleus, where *TBL1X* is expressed only from the active X chromosome, the IF signal intensity is 1140 a.u., whereas in the *right* nucleus, where both Xa and Xi *TBL1X* alleles are expressed, the intensity is as high as 1560 a.u. (*E*) Boxplot shows the levels of TBL1X signal intensity either in the whole cell population (bulk; *left* box) or in cells in which *TBL1X* is expressed only from the active X chromosome (*middle* box) or when *TBL1X* is expressed from all X chromosomes (*right* box). The upper whisker represents the maximum value, upper quartile 75%, median 50%, lower quartile 25%, and lower whisker the minimum value of the data set. Nuclei number analyzed is indicated *above* the *x*-axis. (*F*) Cell sorting of ZR-75-1 cells based on TBL1X signal intensity. An IgG antibody has been used as negative control. (*G*) Bar graph shows the level of *TBL1X* expression from the *XIST*-coated X chromosome by pyrosequencing at SNP rs16985675. *Left* bar represents the gDNA control, which is in agreement with the allelic imbalance (i.e., one Xi allele and two Xa alleles). Data represent the mean values ±SEM. (***) *P* < 0.001; (**) *P* < 0.01; (*) *P* < 0.05 using the Student's *t*-test.

In order to assess allelic expression of specific genes at the single-cell level, we developed RNA FISH probes for several X-linked escapee genes, bypassing the issue of uninformative SNPs. We confirmed that *HDAC8* is expressed from the *XIST* RNA-associated Xi chromosome only in MDA-MB-436 and SK-BR-3 cells (Supplemental Fig. S5D), whereas *TBL1X* was expressed from Xi only in ZR-75-1 (Fig. 5A). We also confirmed that *APOOL* and *SYTL4* are only escaping from XCI in MDA-MB-436 by RNA FISH (data not shown). *ATRX* was used as a control gene that is subject to XCI in all five cell lines (Supplemental Fig. S5E).

We then investigated the degree to which reactivation could impact on gene dosage for *TBL1X*, one of the “cancer-specific” escapees in ZR-75-1 cells. Using IF against TBL1X combined with RNA FISH, we correlated the protein levels of TBL1X to its expression from the Xi (Fig. 5B). On average, in ZR-75-1, the IF signals appear highly heterogeneous but also stronger than the four other cell lines (in agreement with the RNA level) (Fig. 5C; Supplemental Fig. S5F). We noted that MDA-MB-436 cells also showed slightly increased protein levels, consistent with overall *TBL1X* expression levels in this cell line, which must be due to higher expression of the single active allele (on the Xa) in this cell line. To determine whether the higher protein levels in ZR-75-1 are due to reactivation of *TBL1X* on the Xi or to overexpression of the active alleles on the Xa (as in MDA-MB-436), we quantitated the IF signal in cells that do, or do not, show *TBL1X* transcriptional reactivation on the Xi (Fig. 5D). Significantly more TBL1X staining was seen in ZR-75-1 nuclei that displayed *TBL1X* reactivation (Fig. 5E). We also sorted ZR-75-1 cells by FACS based on TBL1X staining intensity (Fig. 5F) and observed significantly more biallelic expression of *TBL1X* in cells with the highest levels of TBL1X protein staining (Fig. 5G).

### Local epigenetic erosion affects genes that escape XCI in cancer

To investigate further the underlying causes of Xi gene reactivation in cancer cells, we investigated the chromatin status of “cancer-specific” escapees at the molecular level. First, the DNA methylation status of multiple X-linked gene promoters was investigated using EpiTYPER analysis (Sequenom) (Supplemental Fig. S6A). All escapees (normal or “cancer-specific”) showed low levels of DNA methylation at their promoters (e.g., *KDM5C*, *HDAC8*). However, we noted that some genes subject to XCI (i.e., only expressed from the Xa) in cancer cell lines, nevertheless showed low promoter methylation (e.g., *TBL1X* in SK-BR-3 cells or *HDAC8* in ZR-75-1). This suggests that they might be more prone to reactivation in a cancer context, with outright reexpression from the Xi in only some cell lines.

**Figure 6.**
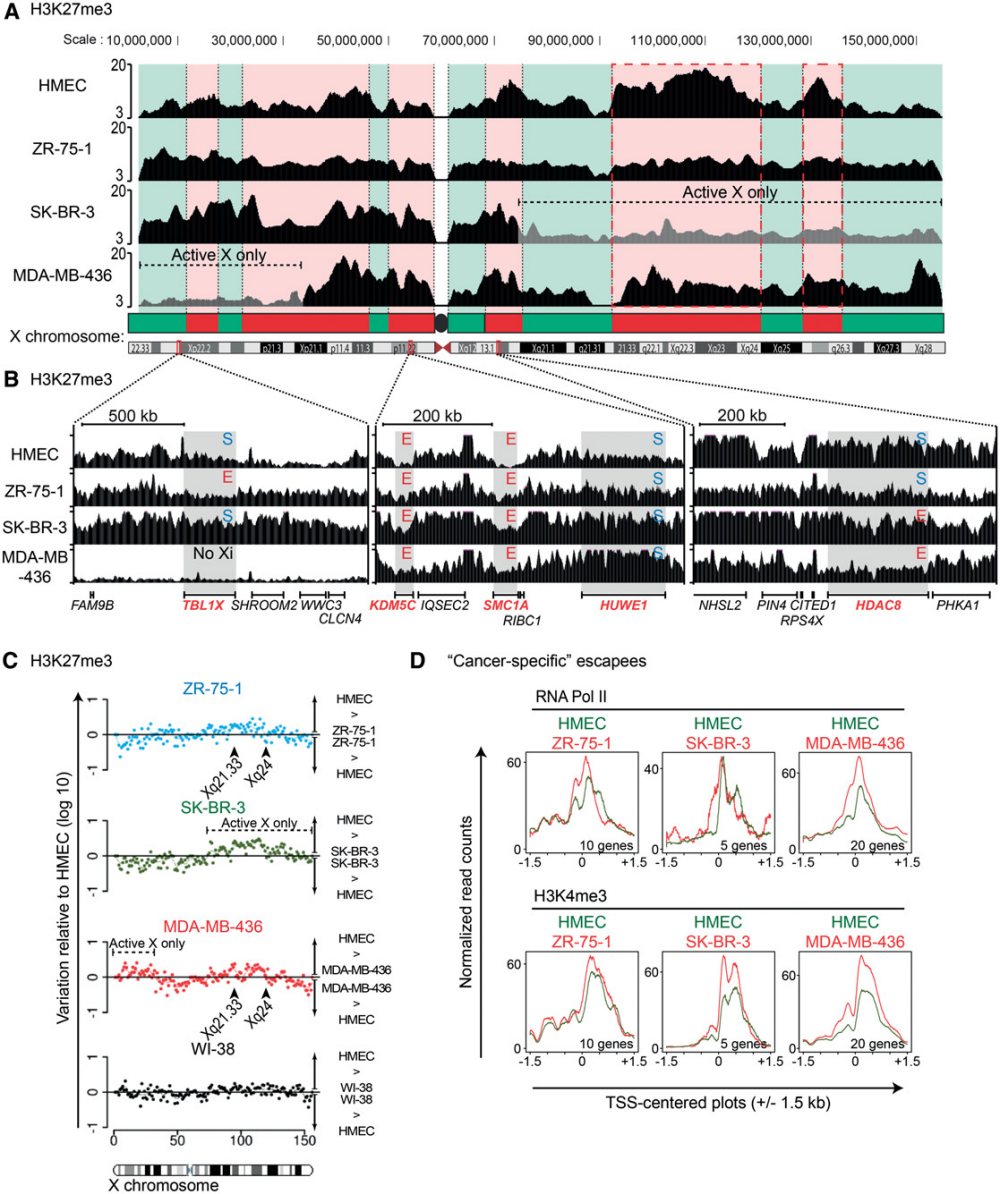
Chromatin landscape of the inactive X chromosome is disrupted in breast cancer cell lines. (*A*) Scheme of H3K27me3 enrichment (ChIP-seq) across the whole X chromosome. Red and green domains represent H3K27me3 and H3K9me3 enriched regions, respectively, as identified in normal human cells (Chadwick 2007). Regional loss of inactive X is indicated (and depicted by gray region). The two main enriched H3K27me3 domains’ loss in ZR-75-1 and MDA-MB-436 are depicted by the two red dotted rectangles. (*B*) H3K27me3 enrichment is detailed for three regions of the X chromosome carrying genes subjected (S) or escaping XCI (E). (*C*) Dot plots show variation of H3K27me3 enrichment along the X chromosome (1-Mb bins) of the three tumoral cell lines and WI-38 relative to HMEC. (*D*) TSS-centered plots (±1.5 kb) show RNA Pol II and H3K4me3 enrichment for the “cancer-specific” escapees (cf. Supplemental Table S1) of each tumoral cell line (red line) and HMEC (green line). The number of genes analyzed is indicated *below* each plot.

We also performed chromatin immunoprecipitation and sequencing (ChIP-seq) on normal and cancer cell lines to assess Xi chromatin status. We investigated H3K27me3 (associated with the inactive state of the Xi), H3K4me3 (enriched at transcriptional start sites [TSSs] of active genes), and RNA Pol II. The comparison of quantile-normalized H3K27me3 profiles revealed major changes for the X chromosomes between normal and tumor cells (Fig. 6A). In HMECs, low-resolution chromosome-wide profiles exhibited a pattern of domains that is highly reminiscent of the distinct nonoverlapping regions of the human Xi previously reported for H3K9me3 and H3K27me3 (Chadwick 2007; Chadwick and Willard 2004). Indeed, comparing the H3K9me3 and H3K27me3 data from the ENCODE Project Consortium (2012) with our H3K27me3 ChIP-seq data sets, these different types of heterochromatin domains are readily detectable in normal HMEC and WI-38 cells (Supplemental Fig. S6C; data not shown). In contrast, the organization of these H3K27me3-enriched domains was found to be heavily perturbed in ZR-75-1 and MDA-MB-436. In ZR-75-1 cells, the X chromosome displays a global, nearly uniform pattern of H3K27me3, with no discernable enriched domains (Fig. 6A). The analyzable parts of the X in SK-BR-3 cells (where an Xi is retained) are much less perturbed, apparently respecting the H3K27me3 domains. These results are in line with the reorganization of the Xi in interphase cells by IF/FISH (Fig. 2A–C). The X chromosome in MDA-MB-436 shows a heavily segmented H3K27 methylation profile, as (1) the beginning of the short arm shows no H3K27me3 marks (evident consequence of the loss of the Xi fragment); (2) the rest of the short arm displays significant H3K27me3 enrichment, although the profile is rather different from that seen for HMEC; (3) the region surrounding the XIC shows a profile similar to that seen in normal cells; and (4) the region spanning Xq21.33 to the end of the long arm, which is no longer linked to the XIC (see Fig. 3), does not display discernable H3K27me3 domains; in particular, the two highly enriched domains visible in normal cells are lacking (Fig. 6A, red dotted rectangles). To further consolidate these observations, we compared the variation of H3K27me3 signals along the X chromosome between HMEC and the other four cell lines (WI-38 and the three tumor cell lines). Highly variable H3K27me3 patterns across the X chromosome were observed in the tumor cell lines, and several regions for which an Xi copy was still present showed a drastic decrease in H3K27me3 levels (e.g., the Xq21.33-Xq24 region in ZR-75-1 and MDA-MB-436) (Fig. 6C). On the other hand, much less pronounced variation in H3K27me3 distributions on the Xi was observed when HMEC and WI-38 cells were compared, despite their divergent tissue origins (lung fibroblasts versus mammary epithelial cells) (Fig. 6C). Importantly, in the breast cancer lines, the perturbations were not unique to the Xi, as we also noted aberrant H3K27me3 landscapes across autosomal regions of cancer cells (e.g., Chromosome 17 on Supplemental Fig. S6B), indicating that this is a genome-wide characteristic of tumor cells. Thus, we conclude that both genome-wide and Xi-specific distributions of H3K27me3 are severely disrupted in breast tumor cell lines. Although this is partly due to genetic changes (Xi translocations and regional losses), the Xi epigenomic landscape is clearly disorganized, consistent with our aforementioned observations using IF.

Next, we assessed patterns of H3K4me3 and RNA Pol II around the TSS of X-linked genes, and noted that the escapees identified in each cell line displayed a generally higher enrichment of RNA Pol II and H3K4me3 than X-linked genes that were expressed only from the Xa (Supplemental Fig. S6D,E). Similarly, “cancer-specific” escapees generally exhibited higher enrichment at their TSS in the cell lines where they escaped compared to HMECs (Fig. 6D; Supplemental Fig. S7A) with a few exceptions (e.g., *CFP*, *FLNA*, and *MOSPD1* in MDA-MB-436 cells displayed no obvious differences in TSS profiles) (Supplemental Fig. S7B). We also noted that “cancer-specific” escapees, such as *HDAC8* or *NXT2*, exhibit additional and/or enlarged H3K4me3 sites in tumor cells when compared to HMEC (Supplemental Fig. S7B).

As H3K27me3 is normally rather broadly distributed on the Xi, rather than being TSS centered (Marks et al. 2009; Simon et al. 2013), we examined the local environment of genes that normally escape XCI (e.g., *KDM5C* and *SMC1A*) or are silenced on the Xi (e.g., *HUWE1*) and found them to display the expected low and high enrichments, respectively (Fig. 6B, center panel). For “cancer-specific” escapees (*TBL1X* in ZR-75-1, *HDAC8* in MDA-MB-436 and SK-BR-3), no obvious systematic correlation between local H3K27me3 levels and escape/silencing could be seen (Fig. 6B, left and right panels). Although the global disorganization of H3K27me3 domains in tumor cell lines is not necessarily reflected locally at the level of genes, H3K27me3 disorganization may nevertheless affect long-range regulatory landscapes, creating a context favoring escape in concert with additional events.

Finally, we monitored allele-specific enrichment of H3K4me3, RNA Pol II peaks, and H3K27me3 enrichment across genes with informative SNPs. *HUWE1* revealed exclusively monoallelic enrichment for all three marks, consistent with its silence on the Xi in all lines (Supplemental Fig. S7C), whereas escapees *SMC1A* and *DDX3X* and several “cancer-specific” escapees displayed biallelic H3K4me3 and RNA Pol II, with monoallelic H3K27me3 (Supplemental Fig. S7C,D). Thus, for informative escapees in the three cancer cell lines, H3K27me3 is observed on one allele, whereas both alleles show signs of active transcription (H3K4me3 and RNA Pol II occupancy).

### Perturbation of the inactive X chromosome is also found in primary breast tumors

**Figure 7.**
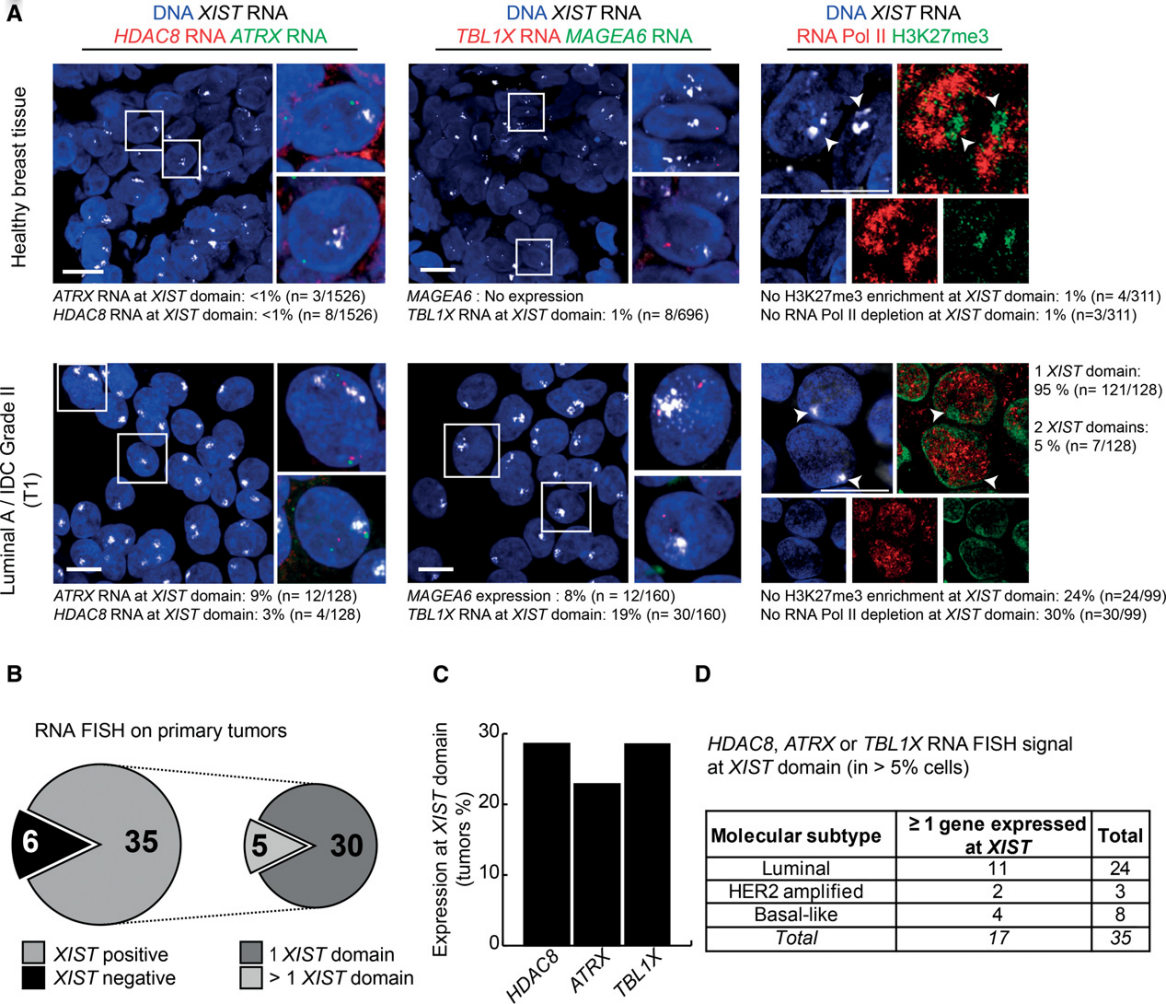
The inactive X chromosome is reactivated in primary breast tumors. (*A*) Z-projections of 3D RNA FISH show representative examples of expression of *HDAC8* (red) and *ATRX* (green) (*left*) or *TBL1X* (red) and *MAGEA6* (green) (*middle*) at *XIST* domains (gray) in healthy breast tissue and invasive ductal carcinoma (IDC; Luminal A Grade III tumor). On the *right*, Z-projections of super-resolutive 3D immuno-RNA FISH show representative examples of the level of H3K27me3 enrichment (green) and RNA Pol II depletion (red) on *XIST* RNA domains (gray) in healthy and tumoral breast tissues. Arrowheads indicate the *XIST* domains. Quantification of RNA Pol II exclusion and H3K27me3 enrichment at *XIST* domains have been carried out on images acquired with a confocal spinning-disk microscope. Scale bar, 10 µm. (*B*) Summary of the *XIST* domain positive (domains in >10% of the nuclei) and negative tumors among the 41 primary breast tumors studied. (*C*) Summary of the number of tumors harboring *HDAC8*, *ATRX*, or *TBL1X* expression at *XIST* domain (assessed by RNA FISH). A gene showing expression within the *XIST* domain in >5% of the nuclei is considered as reactivated in this tumor. (*D*) The table recapitulates the number of *XIST* positive tumors with Xi-linked gene reactivation according to their molecular subtypes: Luminal, HER2 amplified, or Basal-like (BCL).

We next assessed whether epigenetic disruption of the Xi also occurs in primary breast tumors. Due to the cellular heterogeneity in such samples, as well as the variable presence of normal stromal cells, we focused on single-cell techniques (IF and RNA FISH) to investigate the Xi. We analyzed seven tumors using a tumor stamp technique with fresh samples (see Methods) to evaluate the degree of enrichment of H3K27me3 at sites of *XIST* RNA accumulation (Fig. 7A; Supplemental Fig. S8). H3K27me3 enrichment on the Xi in tumors was highly variable, showing almost no enrichment in four of the seven tumors analyzed: T1, T2, T4, and T4meta. This confirmed our observations from cell lines that Xi chromatin status is frequently disrupted in breast cancer. We also noted that H3K27me3 enrichment within a *XIST* RNA domain was not necessarily accompanied by a depletion of RNA Pol II (e.g., tumors T1 and T2) (Fig. 7A; Supplemental Fig. S8A). We also noted a significant decrease in DNA enrichment at the level of the *XIST* RNA domain in primary tumors (Supplemental Fig. S8E,F). Taken together, these results demonstrate that the Xi shows significant chromosome disorganization and chromatin disruption in primary breast tumors, similarly to the tumor cell lines described above and that suggesting that disappearance of the Barr body in certain breast cancers is indeed due to epigenetic instability.

We next assessed whether the aberrant chromatin status of the Xi also translated into X-linked gene reactivation by assessing *XIST* together with *HDAC8*, *ATRX*, *MAGEA6*, and *TBL1X* expression on fresh tumor stamps (including those analyzed above) or tumor-tissue cryosections. These genes were chosen because (1) they are robustly detected by RNA FISH; (2) they are “cancer-specific” escapees in some tumor cell lines (except *ATRX* and *MAGEA6*); and (3) *HDAC8* and *ATRX* lie in proximity to each other and to *XIST* (within a few megabases), thus minimizing their chances of being separated by translocations and facilitating RNA FISH analysis in tumors. We analyzed 41 primary breast tumors with corresponding normal tissue for 15 of them (examples shown in Fig. 7A; Supplemental Fig. S8). Thirty-five tumors were *XIST*-positive, with at least one *XIST* RNA domain in ≥10% of nuclei (Fig. 7B). The number, organization, and intensity of *XIST* RNA domains varied substantially between tumors and even among cells of the same tumor (Supplemental Fig. S8). For X-linked genes, aberrant reactivation from the Xi was considered to occur if ≥5% of nuclei harbored a nascent RNA FISH signal at or within a *XIST* RNA domain of a given sample. With these criteria, we found 28%, 20%, and 29% of tumors displayed aberrant *HDAC8*, *ATRX*, and *TBL1X* expression from the inactive X, respectively (Fig. 7C). Note that in healthy breast tissue, we never observed >1% of nuclei showing X-linked gene RNA FISH signal within the *XIST* RNA domain. Furthermore, we did not observe higher degrees of reactivation for any of these three X-linked genes in particular cancer subtypes, although only a limited number of HER2+ and basal-like tumors were analyzed (Fig. 7D). We also analyzed the cancer/testis antigen family 1, member 6 *MAGEA6* gene, which is normally silent on both Xa and Xi. None of the primary tumors showed reactivation from the Xi, although in some tumors, *MAGEA6* expression was detected from the presumed Xa (Fig. 7A; Supplemental Fig. S8B–D), similarly to our data in breast cancer cell lines. In summary, RNA FISH analysis of 35 *XIST*-positive primary breast tumors of the luminal, HER2+, and basal-like subtypes, revealed that all three X-linked genes tested, *HDAC8*, *TBL1X*, and even *ATRX*, show Xi reactivation in a significant proportion of tumor cells in stark contrast to the situation in healthy breast tissue from the same patient.

To extend our findings, we analyzed publicly available data for biallelic expression of X-linked genes, using a data set for which both RNA-seq and DNA SNP6 data were available (Shah et al. 2012). After we filtered out tumors of “poor” quality (see Supplemental Methods) and those contaminated by normal cells (Popova et al. 2009), we identified 25 BLC tumor samples with a heterozygous X chromosome, suggesting they likely retained an inactive X or at least some region of the Xi (Supplemental Fig. S9A; Supplemental Table S2). Among these tumors, we identified 183 informative genes, of which 78 were expressed biallelically and 105 monoallelically. Almost half of these biallelically expressed genes are subject to XCI in healthy human cells (Supplemental Fig. S9B; Cotton et al. 2013). Furthermore, in agreement with our findings in the three tumor cell lines, *TBL1X*, *NXT2*, and *DOCK11* were among the 14 genes that were biallelically expressed in at least two primary breast tumors (Supplemental Fig. S9C). We identified no obvious correlation between the degree of “cancer-specific” escape from XCI and the BRCAness of the tumor (as defined in Popova et al. 2012; Supplemental Table S2).

In summary, our analysis of Xi transcriptional status in a total of about 140 primary breast tumors of the luminal, HER2+, and basal-like subtypes, using both RNA FISH and RNA-seq analyses, revealed that multiple X-linked genes are reactivated on the inactive X chromosome.

## Discussion

We have conducted an in-depth investigation of the nuclear organization, chromatin status, and chromosome-wide transcriptional activity of the inactive X chromosome in breast cancer cell lines and primary tumor samples. We can conclude that a frequent cause of Barr body loss in breast cancer is due to the global perturbation of its nuclear organization and disruption of its heterochromatic structure. Furthermore, the aberrant epigenomic landscapes we have uncovered for the Xi in breast cancer cells are accompanied by a significant degree of sporadic gene reactivation, which in some cases can lead to aberrant dosage at the protein level (Supplemental Fig. S10A).

### Epigenetic erosion of the Barr body in breast cancer

Epigenetic perturbations of the inactive X chromosome were found at multiple levels in breast cancer. Based on microscopy, *XIST* RNA coating was often found to be highly dispersed, with variable H3K27me3 enrichment, and a marked absence of an RNA Pol II-depleted nuclear compartment. Based on ChIP-seq, abnormal presence of both RNA Pol II and H3K4me3 was observed at “cancer-specific” escapees, reminiscent of the chromatin organization of the normally escapees from XCI in noncancer cells (Kucera et al. 2011; Cotton et al. 2013). Importantly, however, virtually all informative “cancer-specific” escapees displayed simultaneously repressive (H3K27me3) and active (H3K4me3, RNA Pol II recruitment) chromatin marks (see Supplemental Fig. S7D), suggestive of bivalent chromatin, as observed in ES cells (Bernstein et al. 2006), which may reflect, or even underlie, metastable states of gene expression from the Xi in a cancer context. The Xi was also severely perturbed at a more global chromatin level, with aberrant distributions of H3K27me3 and acetylation of H3 and H4 present in interphase breast cancer cells. The disruption of H3K27me3 domains that we observed based on ChIP-seq in breast cancer cell lines may reflect the nuclear disorganization of the Xi, as it has been shown that H3K27me3 enriched domains in normal cells tend to be clustered together in interphase and most likely participate in the specific chromosomal and nuclear organization of the Barr body (Chadwick and Willard 2004). Nevertheless, despite these global and local epigenetic perturbations in all the breast cancer cell types examined, the Xi could still be distinguished from the Xa. For example, although the degree of enrichment for H3K27me3 on the Xi is lower in cancer cells when compared to HMEC and WI-38 cells, it is still higher than the mean enrichment found over the rest of the genome (Fig. 2B; Supplemental Fig. S10B). Similarly, although exclusion of Cot-1 RNA, RNA Pol II, and euchromatic marks is not complete on the Xi in cancer samples, some degree of exclusion is nevertheless detectable in a subset of cells. Furthermore, “cancer-specific” escapees (like normal escapees) were never expressed to the same levels as their counterparts on the active X.

### Possible causes of the epigenetic instability of the inactive X chromosome in breast cancer

Epigenetic instability of the Xi appears to occur across a broad spectrum of breast cancer types with no obvious specificities for particular molecular subclasses. For example, elevated genetic instability, such as in *BRCA1* null and basal-like breast tumors (Richardson et al. 2006; Vincent-Salomon et al. 2007) cannot explain the marked epigenetic instability that we found in all subtypes. We believe that the underlying causes of the structural and transcriptional lability of the Xi in cancer are probably a result of both genetic and epigenetic defects. For example, the slightly lower levels of *XIST* expression that we observed in most cases might lead to less efficient chromosome coating and contribute to the disruption of the silent nuclear compartment normally present in somatic cells (Chaumeil et al. 2006; Clemson et al. 2006), as well as to the aberrant distribution of H3K27me3 and other chromatin marks. Furthermore, the precise combination of epigenetic factors that ensure the inactive state of different genes on the inactive X chromosome in somatic cells is still very much an open question. Indeed, our study revealed that the rather global epigenetic misregulation in tumor cells results in rather sporadic X-linked gene reactivation, and escape from silencing may be dependent on a gene's local environment, as neighboring genes can behave very differently in a cancer context. For example, the *NXT2* gene was found to show aberrant transcription, whereas its close neighbor, *NUP62CL*, remained silent in MDA-MB-436 cells, although both lie in a non-*XIST*-coated/H3K27me3 depleted region of the Xi (Supplemental Table S1).

### Consequences of Xi erosion in breast cancer cells

The epigenetic instability of the Xi in breast cancer, which can result in aberrant X-linked gene expression, might in some cases contribute to a selective advantage for cancer cells. Indeed, several “cancer-specific” escapees identified here have previously been shown to be involved in cancer, such as *HDAC8*, which is implicated in cellular transformation (Oehme et al. 2009) and metastasis formation (Park et al. 2011). *TBL1X*, for which we demonstrated increased protein dosage in the context of its aberrant reactivation from the Xi, belongs to a complex with *HDAC3* that is directly linked to several forms of cancer (Spurling et al. 2008; López-Soto et al. 2009; Kim et al. 2010; Müller et al. 2013; Miao et al. 2014). Aberrant dosage of such X-linked chromatin-associated factors could easily be imagined to lead to pleiotropic effects in a cancer context, promoting or enhancing more genome-wide misregulation. Further studies will be required to explore the extent to which X-linked gene reactivation might contribute to cancer progression.

Importantly, in addition to the aberrant reactivation of genes on the inactive X, aberrant silencing of several genes that normally escape XCI, such as *RAB9A*, *BCOR*, *RPL39*, or *PNPLA4* was also observed in tumor cell lines. *BCOR* mutations have already been implicated in some cancers (Zhang et al. 2012). Aberrant repression of such genes in a cancer context might be due to sporadic epimutation or to impaired protection from XCI through perturbation of boundary elements (Filippova et al. 2005). Finally, we also showed that abnormal activation of cancer/testis Antigen genes, which are known to be aberrantly expressed in cancer, was from the active rather than the inactive X chromosome in one case (*MAGEA6*), pointing to differences in the stability of silent genes on the active versus the inactive X chromosomes in cancer.

### Consequences of genetic instability on the epigenetic status of the Xi in cancer cells

Our study also reveals how chromosomal rearrangements, such as deletions or translocations can have an impact on the epigenetic status of a chromosome through loss of the XIC from an inactive X fragment and/or juxtaposition of the XIC to an autosome. We found such a scenario in the MDA-MB-436 cell line, where loss of the XIC from an Xi fragment resulted in reduced H3K27me3 enrichment on the Xi, as expected from previous reports demonstrating that PRC2 is recruited (directly or indirectly) to the Xi via *XIST* RNA (Wutz et al. 2002; Plath et al. 2004; Maenner et al. 2010). However, the H3K27me3 profile on this Xi fragment is not equivalent to a euchromatin region, indicating that other mechanisms may act to maintain an intermediate heterochromatic organization. Furthermore, loss of *XIST* RNA coating and reduced H3K27me3 was not sufficient to result in notably higher rates of sporadic gene reactivation of the inactive X-chromosome fragment when compared to Xi fragments carrying an XIC and expressing *XIST* (Supplemental Fig. S10A). This is presumably because other marks, such as hypoacetylation of H4, hypomethylation of H3K4, and promoter DNA methylation, are not fully perturbed and can propagate the inactive state. Thus, although *XIST* RNA and PRC2-associated chromatin changes may participate in maintaining the inactive state, they do not appear to be essential in the context of this particular cell line. We also made the intriguing observation that in X:autosome translocations involving an Xi fragment still carrying an XIC and expressing *XIST* RNA, H3K27me3 enrichment could be found to spread into the autosomal sequences adjacent to the XIC (for example in the SK-BR-3 and MDA-MB-436 cell lines) (Fig. 3C). Although we were not able to evaluate whether this results in aberrant gene silencing, such a spread of heterochromatin into autosomal regions as previously shown (Cotton et al. 2014) could clearly have important implications in a cancer context by inducing functional LOH for critical genes such as tumor suppressors.

In conclusion, the perturbed transcriptional and chromatin status of the inactive X chromosome that we have identified in the context of breast cancer opens up several important clinical perspectives. Today, there is still no rapid and efficient way to evaluate the epigenetic instability of tumor cells in a clinical context (Portela and Esteller 2010). In theory, detection of X-linked gene reactivation and aberrant chromatin status using IF and RNA FISH in breast tumors could provide valuable biomarkers to assess epigenetic status and/or to evaluate responsiveness of tumors to drug treatments (Huang et al. 2002). Whether the same degree of Xi epigenetic instability will be found in other types of cancer remains an interesting question for the future.

## Methods

### RNA, DNA FISH, and immunofluorescence

For *XIST* RNA FISH, a combination of two probes covering 16 kb of *XIST* mRNA was used (Okamoto et al. 2011). For nascent transcript detection by RNA FISH, the following BAC (CHORI) probes were used: *HDAC8* (RP11-1021B19), *TBL1X* (RP11-451G24), *ATRX* (RP11-42M11), *HUWE1* (RP11-155O24), and *KDM5C* (RP11-258C19). The correct chromosomal location of BACs was first verified using DNA FISH on metaphase spreads. A FISH probe for *MAGEA6* was generated by cloning the genomic sequence in pCR-XL-TOPO vector. Human Cot-1 DNA (Invitrogen) was used for Cot-1 RNA FISH. Probes were labeled by nick translation (Vysis) with Spectrum Red-dUTP, Spectrum Green-dUTP, or Cy5-dUTP following the manufacturer's instructions. RNA and DNA FISH were performed as described previously (Chaumeil et al. 2008). For more details see Supplemental Methods.

### Microscopy

Images were generated using a Nikon confocal spinning disk microscope fitted with a 60×/1.4 OIL DIC N2 PL APO VC objective. For super resolution imaging, structured illumination (3D-SIM) was performed using a DeltaVision OMX microscope (GE Healthcare).

### Human SNP Array 6.0 DNA and nascent RNA experiments

#### DNA copy number profiles

Genomic profiling was performed at Institut Curie using Affymetrix Human SNP Array 6.0; cell files were processed by Genotyping Console 3.0.2 (Affymetrix, reference model file HapMap270, version 29). Human SNP Array 6.0 data were mined using the previously described and validated GAP method (Popova et al. 2009). Segmental absolute copy numbers and allelic contents (major allele counts) were detected. R scripts and full details of the application are available at http://bioinfo-out.curie.fr/projects/snp_gap/ and have been previously reported (Popova et al. 2009). For more details see Supplemental Methods.

#### Nascent RNA allelic expression

Preparation of samples and analysis of nascent RNA were performed as described previously (Gimelbrant et al. 2007). Briefly, we purified nuclei of assessed cell lines (Nuclei Pure Isolation Kit, Sigma) and subsequently purified nuclear RNA (by classical phenol:chlorophorm extraction). Then, we hybridized cDNA obtained by reverse transcription of nuclear RNA of each sample onto Affymetrix Human SNP Array 6.0. Data was normalized by Genotyping console, and raw single-SNP intensities were taken as allelic expression of corresponding genes. Each SNP was characterized by (1) global expression level score; (2) allelic expression ratio score; and (3) genomic status (loss or retention of heterozygosity score), which were summarized into a biallelic and monoallelic expression status. Genome-wide biallelic and monoallelic expression profiles were obtained by cumulating SNP status in a 50-SNP window and at gene level.

### Primary tumors

A hematein-eosin-safran (HES)–stained tissue section was made in each primary tumor to evaluate tumor cellularity and diagnosis. Characterization of the tumor samples was completed by the determination of estrogen receptor, progesterone receptor, ERBB2, cytokeratin 5/6, and epidermal growth factor receptor (EGFR) status determined by immunohistochemistry done according to previously published protocols (Azoulay et al. 2005). All experiments were performed in accordance with the French Bioethics Law 2004-800, the French National Institute of Cancer (INCa) Ethics Charter, and after approval by the Institut Curie review board and the ethics committees of our institution (“Comité de Pilotage of the Groupe Sein”). In the French ethics law, patients gave their approval for the use of their surgical tumor specimens for research. Data were analyzed anonymously.

For details on experimental procedures used for cell culture, DNA methylation analysis, Sanger sequencing, real-time PCR, allele-specific PCR, pyro-sequencing, RNA sequencing analysis, and chromatin immunoprecipitation analysis, see Supplemental Methods.

## Data access

All high-throughput data from this study have been submitted to the NCBI Gene Expression Omnibus (GEO; http://www.ncbi.nlm.nih.gov/geo/) under accession number GSE62907.

## Supplementary Material

Supplemental Material

## References

[CHALIGNEGR185926C1] Agrelo R, Wutz A. 2010 ConteXt of change—X inactivation and disease. EMBO Mol Med 2: 6–15. 20043281 10.1002/emmm.200900053 PMC3377189

[CHALIGNEGR185926C2] Azoulay S, Laé M, Fréneaux P, Merle S, Al Ghuzlan A, Chnecker C, Rosty C, Klijanienko J, Sigal-Zafrani B, Salmon R, 2005 KIT is highly expressed in adenoid cystic carcinoma of the breast, a basal-like carcinoma associated with a favorable outcome. Mod Pathol 18: 1623–1631. 16258515 10.1038/modpathol.3800483

[CHALIGNEGR185926C3] Barr ML, Moore KL. 1957 Chromosomes, sex chromatin, and cancer. Proc Can Cancer Conf 2: 3–16. 13437159

[CHALIGNEGR185926C4] Bennett CL, Christie J, Ramsdell F, Brunkow ME, Ferguson PJ, Whitesell L, Kelly TE, Saulsbury FT, Chance PF, Ochs HD. 2001 The immune dysregulation, polyendocrinopathy, enteropathy, X-linked syndrome (IPEX) is caused by mutations of *FOXP3*. Nat Genet 27: 20–21. 11137993 10.1038/83713

[CHALIGNEGR185926C5] Bernstein BE, Mikkelsen TS, Xie X, Kamal M, Huebert DJ, Cuff J, Fry B, Meissner A, Wernig M, Plath K, 2006 A bivalent chromatin structure marks key developmental genes in embryonic stem cells. Cell 125: 315–326. 16630819 10.1016/j.cell.2006.02.041

[CHALIGNEGR185926C6] Boggs BA, Cheung P, Heard E, Spector DL, Chinault AC, Allis CD. 2002 Differentially methylated forms of histone H3 show unique association patterns with inactive human X chromosomes. Nat Genet 30: 73–76. 11740495 10.1038/ng787

[CHALIGNEGR185926C7] Carone DM, Lawrence JB. 2013 Heterochromatin instability in cancer: from the Barr body to satellites and the nuclear periphery. Semin Cancer Biol 23: 99–108. 22722067 10.1016/j.semcancer.2012.06.008 PMC3500402

[CHALIGNEGR185926C8] Carrel L, Willard HF. 2005 X-inactivation profile reveals extensive variability in X-linked gene expression in females. Nature 434: 400–404. 15772666 10.1038/nature03479

[CHALIGNEGR185926C9] Chadwick BP. 2007 Variation in Xi chromatin organization and correlation of the H3K27me3 chromatin territories to transcribed sequences by microarray analysis. Chromosoma 116: 147–157. 17103221 10.1007/s00412-006-0085-1

[CHALIGNEGR185926C10] Chadwick BP, Willard HF. 2004 Multiple spatially distinct types of facultative heterochromatin on the human inactive X chromosome. Proc Natl Acad Sci 101: 17450–17455. 15574503 10.1073/pnas.0408021101 PMC534659

[CHALIGNEGR185926C11] Chaumeil J, Okamoto I, Guggiari M, Heard E. 2002 Integrated kinetics of X chromosome inactivation in differentiating embryonic stem cells. Cytogenet Genome Res 99: 75–84. 12900548 10.1159/000071577

[CHALIGNEGR185926C12] Chaumeil J, Le Baccon P, Wutz A, Heard E. 2006 A novel role for Xist RNA in the formation of a repressive nuclear compartment into which genes are recruited when silenced. Genes Dev 20: 2223–2237. 16912274 10.1101/gad.380906 PMC1553206

[CHALIGNEGR185926C13] Chaumeil J, Augui S, Chow JC, Heard E. 2008 Combined immunofluorescence, RNA fluorescent in situ hybridization, and DNA fluorescent in situ hybridization to study chromatin changes, transcriptional activity, nuclear organization, and X-chromosome inactivation. Methods Mol Biol 463: 297–308. 18951174 10.1007/978-1-59745-406-3_18

[CHALIGNEGR185926C14] Chow JC, Heard E. 2010 Nuclear organization and dosage compensation. Cold Spring Harb Perspect Biol 2: a000604. 20943757 10.1101/cshperspect.a000604 PMC2964184

[CHALIGNEGR185926C15] Chow JC, Ciaudo C, Fazzari MJ, Mise N, Servant N, Glass JL, Attreed M, Avner P, Wutz A, Barillot E, 2010 LINE-1 activity in facultative heterochromatin formation during X chromosome inactivation. Cell 141: 956–969. 20550932 10.1016/j.cell.2010.04.042

[CHALIGNEGR185926C16] Clemson CM, Hall LL, Byron M, McNeil J, Lawrence JB. 2006 The X chromosome is organized into a gene-rich outer rim and an internal core containing silenced nongenic sequences. Proc Natl Acad Sci 103: 7688–7693. 16682630 10.1073/pnas.0601069103 PMC1472506

[CHALIGNEGR185926C17] Cotton AM, Ge B, Light N, Adoue V, Pastinen T, Brown CJ. 2013 Analysis of expressed SNPs identifies variable extents of expression from the human inactive X chromosome. Genome Biol 14: R122. 24176135 10.1186/gb-2013-14-11-r122 PMC4053723

[CHALIGNEGR185926C18] Cotton AM, Chen CY, Lam LL, Wasserman WW, Kobor MS, Brown CJ. 2014 Spread of X-chromosome inactivation into autosomal sequences: role for DNA elements, chromatin features and chromosomal domains. Hum Mol Genet 23: 1211–1223. 24158853 10.1093/hmg/ddt513 PMC4051349

[CHALIGNEGR185926C19] Csankovszki G, Panning B, Bates B, Pehrson JR, Jaenisch R. 1999 Conditional deletion of *Xist* disrupts histone macroH2A localization but not maintenance of X inactivation. Nat Genet 22: 323–324. 10431231 10.1038/11887

[CHALIGNEGR185926C20] Csankovszki G, Nagy A, Jaenisch R. 2001 Synergism of Xist RNA, DNA methylation, and histone hypoacetylation in maintaining X chromosome inactivation. J Cell Biol 153: 773–784. 11352938 10.1083/jcb.153.4.773 PMC2192370

[CHALIGNEGR185926C21] De Carvalho DD, Sharma S, You JS, Su SF, Taberlay PC, Kelly TK, Yang X, Liang G, Jones PA. 2012 DNA methylation screening identifies driver epigenetic events of cancer cell survival. Cancer Cell 21: 655–667. 22624715 10.1016/j.ccr.2012.03.045 PMC3395886

[CHALIGNEGR185926C22] Elsheikh SE, Green AR, Rakha EA, Powe DG, Ahmed RA, Collins HM, Soria D, Garibaldi JM, Paish CE, Ammar AA, 2009 Global histone modifications in breast cancer correlate with tumor phenotypes, prognostic factors, and patient outcome. Cancer Res 69: 3802–3809. 19366799 10.1158/0008-5472.CAN-08-3907

[CHALIGNEGR185926C23] The ENCODE Project Consortium. 2012 An integrated encyclopedia of DNA elements in the human genome. Nature 489: 57–74. 22955616 10.1038/nature11247 PMC3439153

[CHALIGNEGR185926C24] Filippova GN, Cheng MK, Moore JM, Truong JP, Hu YJ, Nguyen DK, Tsuchiya KD, Disteche CM. 2005 Boundaries between chromosomal domains of X inactivation and escape bind CTCF and lack CpG methylation during early development. Dev Cell 8: 31–42. 15669143 10.1016/j.devcel.2004.10.018

[CHALIGNEGR185926C25] Ganesan S, Silver DP, Greenberg RA, Avni D, Drapkin R, Miron A, Mok SC, Randrianarison V, Brodie S, Salstrom J, 2002 BRCA1 supports XIST RNA concentration on the inactive X chromosome. Cell 111: 393–405. 12419249 10.1016/s0092-8674(02)01052-8

[CHALIGNEGR185926C26] Gendrel AV, Tang YA, Suzuki M, Godwin J, Nesterova TB, Greally JM, Heard E, Brockdorff N. 2013 Epigenetic functions of Smchd1 repress gene clusters on the inactive X chromosome and on autosomes. Mol Cell Biol 33: 3150–3165. 23754746 10.1128/MCB.00145-13 PMC3753908

[CHALIGNEGR185926C27] Gimelbrant A, Hutchinson JN, Thompson BR, Chess A. 2007 Widespread monoallelic expression on human autosomes. Science 318: 1136–1140. 18006746 10.1126/science.1148910

[CHALIGNEGR185926C28] Grigoriadis A, Caballero OL, Hoek KS, da Silva L, Chen YT, Shin SJ, Jungbluth AA, Miller LD, Clouston D, Cebon J, 2009 CT-X antigen expression in human breast cancer. Proc Natl Acad Sci 106: 13493–13498. 19651608 10.1073/pnas.0906840106 PMC2716388

[CHALIGNEGR185926C29] Heard E, Rougeulle C, Arnaud D, Avner P, Allis CD, Spector DL. 2001 Methylation of histone H3 at Lys-9 is an early mark on the X chromosome during X inactivation. Cell 107: 727–738. 11747809 10.1016/s0092-8674(01)00598-0

[CHALIGNEGR185926C30] Huang S, Deerinck TJ, Ellisman MH, Spector DL. 1997 The dynamic organization of the perinucleolar compartment in the cell nucleus. J Cell Biol 137: 965–974. 9166399 10.1083/jcb.137.5.965 PMC2136227

[CHALIGNEGR185926C31] Huang KC, Rao PH, Lau CC, Heard E, Ng SK, Brown C, Mok SC, Berkowitz RS, Ng SW. 2002 Relationship of *XIST* expression and responses of ovarian cancer to chemotherapy. Mol Cancer Ther 1: 769–776. 12492109

[CHALIGNEGR185926C32] Kawakami T, Zhang C, Taniguchi T, Kim CJ, Okada Y, Sugihara H, Hattori T, Reeve AE, Ogawa O, Okamoto K. 2004 Characterization of loss-of-inactive X in Klinefelter syndrome and female-derived cancer cells. Oncogene 23: 6163–6169. 15195139 10.1038/sj.onc.1207808

[CHALIGNEGR185926C33] Keohane AM, O'Neill LP, Belyaev ND, Lavender JS, Turner BM. 1996 X-Inactivation and histone H4 acetylation in embryonic stem cells. Dev Biol 180: 618–630. 8954732 10.1006/dbio.1996.0333

[CHALIGNEGR185926C34] Kim HC, Choi KC, Choi HK, Kang HB, Kim MJ, Lee YH, Lee OH, Lee J, Kim YJ, Jun W, 2010 HDAC3 selectively represses CREB3-mediated transcription and migration of metastatic breast cancer cells. Cell Mol Life Sci 67: 3499–3510. 20473547 10.1007/s00018-010-0388-5 PMC11115716

[CHALIGNEGR185926C35] Kucera KS, Reddy TE, Pauli F, Gertz J, Logan JE, Myers RM, Willard HF. 2011 Allele-specific distribution of RNA polymerase II on female X chromosomes. Hum Mol Genet 20: 3964–3973. 21791549 10.1093/hmg/ddr315 PMC3177651

[CHALIGNEGR185926C36] López-Soto A, Folgueras AR, Seto E, Gonzalez S. 2009 HDAC3 represses the expression of NKG2D ligands ULBPs in epithelial tumour cells: potential implications for the immunosurveillance of cancer. Oncogene 28: 2370–2382. 19430493 10.1038/onc.2009.117

[CHALIGNEGR185926C37] Lyon MF. 1961 Gene action in the *X*-chromosome of the mouse (*Mus musculus* L.). Nature 190: 372–373. 13764598 10.1038/190372a0

[CHALIGNEGR185926C38] Maenner S, Blaud M, Fouillen L, Savoye A, Marchand V, Dubois A, Sanglier-Cianférani S, Van Dorsselaer A, Clerc P, Avner P, 2010 2-D structure of the A region of Xist RNA and its implication for PRC2 association. PLoS Biol 8: e1000276. 20052282 10.1371/journal.pbio.1000276 PMC2796953

[CHALIGNEGR185926C39] Marks H, Chow JC, Denissov S, Françoijs KJ, Brockdorff N, Heard E, Stunnenberg HG. 2009 High-resolution analysis of epigenetic changes associated with X inactivation. Genome Res 19: 1361–1373. 19581487 10.1101/gr.092643.109 PMC2720191

[CHALIGNEGR185926C40] Miao LJ, Huang FX, Sun ZT, Zhang RX, Huang SF, Wang J. 2014 Stat3 inhibits Beclin 1 expression through recruitment of HDAC3 in nonsmall cell lung cancer cells. Tumour Biol 35: 7097–7103. 24760274 10.1007/s13277-014-1961-6

[CHALIGNEGR185926C41] Müller BM, Jana L, Kasajima A, Lehmann A, Prinzler J, Budczies J, Winzer KJ, Dietel M, Weichert W, Denkert C. 2013 Differential expression of histone deacetylases HDAC1, 2 and 3 in human breast cancer—overexpression of HDAC2 and HDAC3 is associated with clinicopathological indicators of disease progression. BMC Cancer 13: 215. 23627572 10.1186/1471-2407-13-215 PMC3646665

[CHALIGNEGR185926C42] Nakagawa M, Oda Y, Eguchi T, Aishima S, Yao T, Hosoi F, Basaki Y, Ono M, Kuwano M, Tanaka M, 2007 Expression profile of class I histone deacetylases in human cancer tissues. Oncol Rep 18: 769–774. 17786334

[CHALIGNEGR185926C43] O'Donovan PJ, Livingston DM. 2010 BRCA1 and BRCA2: breast/ovarian cancer susceptibility gene products and participants in DNA double-strand break repair. Carcinogenesis 31: 961–967. 20400477 10.1093/carcin/bgq069

[CHALIGNEGR185926C44] Oehme I, Deubzer HE, Wegener D, Pickert D, Linke JP, Hero B, Kopp-Schneider A, Westermann F, Ulrich SM, von Deimling A, 2009 Histone deacetylase 8 in neuroblastoma tumorigenesis. Clin Cancer Res 15: 91–99. 19118036 10.1158/1078-0432.CCR-08-0684

[CHALIGNEGR185926C45] Okamoto I, Patrat C, Thépot D, Peynot N, Fauque P, Daniel N, Diabangouaya P, Wolf JP, Renard JP, Duranthon V, 2011 Eutherian mammals use diverse strategies to initiate X-chromosome inactivation during development. Nature 472: 370–374. 21471966 10.1038/nature09872

[CHALIGNEGR185926C46] Pageau GJ, Hall LL, Ganesan S, Livingston DM, Lawrence JB. 2007 The disappearing Barr body in breast and ovarian cancers. Nat Rev Cancer 7: 628–633. 17611545 10.1038/nrc2172

[CHALIGNEGR185926C47] Park SY, Jun JA, Jeong KJ, Heo HJ, Sohn JS, Lee HY, Park CG, Kang J. 2011 Histone deacetylases 1, 6 and 8 are critical for invasion in breast cancer. Oncol Rep 25: 1677–1681. 21455583 10.3892/or.2011.1236

[CHALIGNEGR185926C48] Perou CM, Sørlie T, Eisen MB, van de Rijn M, Jeffrey SS, Rees CA, Pollack JR, Ross DT, Johnsen H, Akslen LA, 2000 Molecular portraits of human breast tumours. Nature 406: 747–752. 10963602 10.1038/35021093

[CHALIGNEGR185926C49] Perry M. 1972 Evaluation of breast tumour sex chromatin (Barr body) as an index of survival and response to pituitary ablation. Br J Surg 59: 731–734. 5070138 10.1002/bjs.1800590912

[CHALIGNEGR185926C50] Plath K, Talbot D, Hamer KM, Otte AP, Yang TP, Jaenisch R, Panning B. 2004 Developmentally regulated alterations in Polycomb repressive complex 1 proteins on the inactive X chromosome. J Cell Biol 167: 1025–1035. 15596546 10.1083/jcb.200409026 PMC2172612

[CHALIGNEGR185926C51] Popova T, Manie E, Stoppa-Lyonnet D, Rigaill G, Barillot E, Stern MH. 2009 Genome Alteration Print (GAP): a tool to visualize and mine complex cancer genomic profiles obtained by SNP arrays. Genome Biol 10: R128. 19903341 10.1186/gb-2009-10-11-r128 PMC2810663

[CHALIGNEGR185926C52] Popova T, Manié E, Rieunier G, Caux-Moncoutier V, Tirapo C, Dubois T, Delattre O, Sigal-Zafrani B, Bollet M, Longy M, 2012 Ploidy and large-scale genomic instability consistently identify basal-like breast carcinomas with *BRCA1/2* inactivation. Cancer Res 72: 5454–5462. 22933060 10.1158/0008-5472.CAN-12-1470

[CHALIGNEGR185926C53] Portela A, Esteller M. 2010 Epigenetic modifications and human disease. Nat Biotechnol 28: 1057–1068. 20944598 10.1038/nbt.1685

[CHALIGNEGR185926C54] Richardson AL, Wang ZC, De Nicolo A, Lu X, Brown M, Miron A, Liao X, Iglehart JD, Livingston DM, Ganesan S. 2006 X chromosomal abnormalities in basal-like human breast cancer. Cancer Cell 9: 121–132. 16473279 10.1016/j.ccr.2006.01.013

[CHALIGNEGR185926C55] Rivera MN, Kim WJ, Wells J, Driscoll DR, Brannigan BW, Han M, Kim JC, Feinberg AP, Gerald WL, Vargas SO, 2007 An X chromosome gene, *WTX*, is commonly inactivated in Wilms tumor. Science 315: 642–645. 17204608 10.1126/science.1137509

[CHALIGNEGR185926C56] Schenk T, Chen WC, Göllner S, Howell L, Jin L, Hebestreit K, Klein HU, Popescu AC, Burnett A, Mills K, 2012 Inhibition of the LSD1 (KDM1A) demethylase reactivates the all-*trans*-retinoic acid differentiation pathway in acute myeloid leukemia. Nat Med 18: 605–611. 22406747 10.1038/nm.2661 PMC3539284

[CHALIGNEGR185926C57] Shah SP, Roth A, Goya R, Oloumi A, Ha G, Zhao Y, Turashvili G, Ding J, Tse K, Haffari G, 2012 The clonal and mutational evolution spectrum of primary triple-negative breast cancers. Nature 486: 395–399. 22495314 10.1038/nature10933 PMC3863681

[CHALIGNEGR185926C58] Shen H, Laird PW. 2013 Interplay between the cancer genome and epigenome. Cell 153: 38–55. 23540689 10.1016/j.cell.2013.03.008 PMC3648790

[CHALIGNEGR185926C59] Silver DP, Dimitrov SD, Feunteun J, Gelman R, Drapkin R, Lu SD, Shestakova E, Velmurugan S, Denunzio N, Dragomir S, 2007 Further evidence for BRCA1 communication with the inactive X chromosome. Cell 128: 991–1002. 17350581 10.1016/j.cell.2007.02.025

[CHALIGNEGR185926C60] Simon MD, Pinter SF, Fang R, Sarma K, Rutenberg-Schoenberg M, Bowman SK, Kesner BA, Maier VK, Kingston RE, Lee JT. 2013 High-resolution Xist binding maps reveal two-step spreading during X-chromosome inactivation. Nature 504: 465–469. 24162848 10.1038/nature12719 PMC3904790

[CHALIGNEGR185926C61] Sirchia SM, Ramoscelli L, Grati FR, Barbera F, Coradini D, Rossella F, Porta G, Lesma E, Ruggeri A, Radice P, 2005 Loss of the inactive X chromosome and replication of the active X in BRCA1-defective and wild-type breast cancer cells. Cancer Res 65: 2139–2146. 15781624 10.1158/0008-5472.CAN-04-3465

[CHALIGNEGR185926C62] Smethurst M, Bishun NP, Fernandez D, Allen J, Burn JI, Alaghband-Zadeh J, Williams DC. 1981 Steroid hormone receptors and sex chromatin frequency in breast cancer. J Endocrinol Invest 4: 455–457. 7334186 10.1007/BF03348311

[CHALIGNEGR185926C63] Spatz A, Borg C, Feunteun J. 2004 X-chromosome genetics and human cancer. Nat Rev Cancer 4: 617–629. 15286741 10.1038/nrc1413

[CHALIGNEGR185926C64] Splinter E, de Wit E, Nora EP, Klous P, van de Werken HJ, Zhu Y, Kaaij LJ, van Ijcken W, Gribnau J, Heard E, 2011 The inactive X chromosome adopts a unique three-dimensional conformation that is dependent on Xist RNA. Genes Dev 25: 1371–1383. 21690198 10.1101/gad.633311 PMC3134081

[CHALIGNEGR185926C65] Spurling CC, Godman CA, Noonan EJ, Rasmussen TP, Rosenberg DW, Giardina C. 2008 HDAC3 overexpression and colon cancer cell proliferation and differentiation. Mol Carcinog 47: 137–147. 17849419 10.1002/mc.20373

[CHALIGNEGR185926C66] Turner NC, Reis-Filho JS. 2006 Basal-like breast cancer and the BRCA1 phenotype. Oncogene 25: 5846–5853. 16998499 10.1038/sj.onc.1209876

[CHALIGNEGR185926C67] Vincent-Salomon A, Ganem-Elbaz C, Manié E, Raynal V, Sastre-Garau X, Stoppa-Lyonnet D, Stern MH, Heard E. 2007 X inactive–specific transcript RNA coating and genetic instability of the X chromosome in *BRCA1* breast tumors. Cancer Res 67: 5134–5140. 17545591 10.1158/0008-5472.CAN-07-0465

[CHALIGNEGR185926C68] Wutz A, Rasmussen TP, Jaenisch R. 2002 Chromosomal silencing and localization are mediated by different domains of *Xist* RNA. Nat Genet 30: 167–174. 11780141 10.1038/ng820

[CHALIGNEGR185926C69] Xiao C, Sharp JA, Kawahara M, Davalos AR, Difilippantonio MJ, Hu Y, Li W, Cao L, Buetow K, Ried T, 2007 The *XIST* noncoding RNA functions independently of BRCA1 in X inactivation. Cell 128: 977–989. 17350580 10.1016/j.cell.2007.01.034

[CHALIGNEGR185926C70] Yildirim E, Kirby JE, Brown DE, Mercier FE, Sadreyev RI, Scadden DT, Lee JT. 2013 Xist RNA is a potent suppressor of hematologic cancer in mice. Cell 152: 727–742. 23415223 10.1016/j.cell.2013.01.034 PMC3875356

[CHALIGNEGR185926C71] Zhang J, Benavente CA, McEvoy J, Flores-Otero J, Ding L, Chen X, Ulyanov A, Wu G, Wilson M, Wang J, 2012 A novel retinoblastoma therapy from genomic and epigenetic analyses. Nature 481: 329–334. 22237022 10.1038/nature10733 PMC3289956

[CHALIGNEGR185926C72] Zink D, Fischer AH, Nickerson JA. 2004 Nuclear structure in cancer cells. Nat Rev Cancer 4: 677–687. 15343274 10.1038/nrc1430

