# Supplementary material for: The inactive X chromosome is epigenetically unstable and transcriptionally labile in breast cancer
Source: Genome Res. 2015 Apr;25(4):488–503. doi: 10.1101/gr.185926.114 (PMC4381521; doi:10.1101/gr.185926.114)
Supplement: Supplemental Material [file supp_25_4_488__index.html]

The inactive X chromosome is epigenetically unstable and transcriptionally labile in breast cancer — The inactive X chromosome is epigenetically unstable and transcriptionally labile in breast cancer — Supplemental Material 

# The inactive X chromosome is epigenetically unstable and transcriptionally labile in breast cancer

## Supplemental Material

**Files in this Data Supplement:**

- Supplemental Material.pdf
- TableS1.xlsx
- TableS2.xlsx
- TableS3.docx
