# Supplementary material for: The inactive X chromosome is epigenetically unstable and transcriptionally labile in breast cancer
Source: Genome Res. 2015 Apr;25(4):488–503. doi: 10.1101/gr.185926.114 (PMC4381521; doi:10.1101/gr.185926.114)
Supplement: Supplemental Material [file supp_gr.185926.114_Supplemental_Material.pdf]

Supplemental Information

The inactive X chromosome is epigenetically unstable and transcriptionally labile in breast cancer

Ronan Chaligné[1,2,3,8], Tatiana Popova[1,4], Marco-Antonio Mendoza-Parra[5], Mohamed-Ashick M. Saleem[5] , David Gentien[1,6], Kristen Ban[1,2,3,8], Tristan Piolot[1,7], Olivier Leroy[1,7], Odette Mariani[6], Hinrich Gronemeyer*[5], Anne Vincent-Salomon*[1,4,6,8], Marc-Henri Stern*[1,4,6]
and Edith Heard*[1,2,3,8]

Extended Experimental Procedures

Cell Culture

Human Mammary Epithelial Cells (HMEC, Invitrogen) were grown in serum-free medium (HuMEC, Invitrogen). WI-38, ZR-75-1, SK-BR-3 and MDA-MB-436 cells were grown in Dulbecco's modified Eagle's medium (DMEM; Invitrogen) containing 10% fetal bovine serum (FBS).

DNA Methylation analysis.

We bisulfite-treated 2 µg of genomic DNA using Epitect bisulfite kit (Qiagen). Bisulfite converted DNA was amplified with bisulfite primers listed in Table S3. All primers incorporated a T7 promoter tag, and PCR conditions are available upon request. We analyzed PCR products by MALDI-TOF mass spectrometry after in vitro transcription and specific cleavage (EpiTYPER by Sequenom®). For each amplicon, we analyzed two independent DNA samples and several CG sites in the CpG Island. Design of primers and selection of best promoter region to assess (approx. 500 bp) were done by a combination of UCSC Genome Browser (http://genome.ucsc.edu) and MethPrimer (http://www.urogene.org). All the primers used are listed (Table S3). NB: MAGEC2 CpG analysis have been done with a combination of two CpG island identified in the gene core.

Analysis of RNA allelic expression profiles (based on Human SNP Array 6.0)

DNA and RNA hybridizations were normalized by Genotyping console. Based on Log2ratios and Allelic Differences of DNA profile absolute segmental copy numbers were inferred. Single SNP raw intensities were considered from RNA profiles. Allelic expression scores were calculated based on both DNA and RNA profiles, as follows:

1. RNA expression of SNP (log(Signal A +Signal B)) was smoothed using a 5 SNPs sliding window and excluding low expressed SNPs (log(Signal A +Signal B)<6.5). Each SNP expression score was calculated by subtracting median and normalizing by standard deviation of expression level shown by exonic and intronic SNPs. Parameters were chosen based on comparisons to mRNA expression profile measured by Affymetrix 133plus2 array.
2. Each SNP homozygosity score was calculated based on the inferred segmental copy number and major allele counts. If the segment was annotated to have a homozygous allelic status, all SNPs from the segment were annotated to be homozygous. If the segment was annotated to have a heterozygous allelic status, Allelic Difference was centered to the median of heterozygous band and normalized by standard deviation of heterozygous band. Homozygosity score of each SNP was set to centered and normalized Allelic Difference.
3. Each SNP allelic expression ratio score was calculated as 2tangent(α) where α corresponded to the angle defined by (Signal A; Signal B) vector (α = 2*arctan(Signal A/Signal B)-pi/2). Balanced allelic expression corresponded to 0.
4. Based on three thresholds: (1) Total expression score, (2) Heterozygous DNA call, (3) Allelic expression ratio score, all SNPs were classified into 6 groups:
    1) No expression or non-informative: designated by 0
    2) Contradictory 1 (homozygous SNP and bi-allelic expression): designated by -1
    3) Contradictory 2 (homozygous AA SNP and mono-allelic BB expression or vice versa): designated by -2
    4) Mono-allelic expression: designated by 1
    5) Bi-allelic expression: designated by 2
    6) Marginal call (in-between mono-allelic and bi-allelic): designated by 1.5

Further analysis and quality controls showed good correspondence between attributions and low number of contradictory calls.

5. Profile of bi-allelic expression was obtained by summarizing bi-allelic expression calls in a sliding window of 50 SNPs.

5bis. Single SNP classification was summarized on the gene level, and each gene allelic expression has been score based on:

1) On the number of informative SNPs in the gene core (belonging to group 4, 5 or 6, see above)

2) On confidence attributed to the SNPs (depending on the distance from the threshold)

3) Consistence between SNPs within the same gene

Sanger sequencing, real-time PCR, allele-specific PCR and pyro-sequencing

Total RNA was isolated from cells using Trizol Reagent (Invitrogen) and purified on columns combined with DNase-treated (Qiagen) to remove contaminating DNA. First-strand cDNA was prepared from 5 µg of RNA and random hexamers using Superscript III (Invitrogen) at 50°C for 1 hr. gDNA was isolated using DNazol reagent (Invitrogen). cDNA and gDNA genotyping status (i.e. single variable position) was determined: either by a real-time PCR single nucleotide polymorphism (SNP) detection system with fluorescent competitive probes using an ViiA7 analyzer (Applied Biosystems), by Sanger sequencing (3130xl Genetic Analyzer, Applied Biosystems) of purified PCR product using BigDye V3.1 kit as recommended by the provider (Applied Biosystems) or by pyrosequencing as recommended by the manufacturer (Qiagen, Pyromark Q24). All real-time PCR reactions used SybrGreen Master Mix (Applied Biosystems) to a final volume of 10 µl. Each sample was analyzed at least in triplicate. All the primers used are listed (Table S3).

RNA sequencing analysis

We performed RNA-sequencing and DNA exome-sequencing on ZR-75-1, SK-BR-3 and MDA-MB-436 cell lines. The RNA-sequencing correspond of paired-reads lane 2x100 bp sequencing on poly-A RNA purified. The DNA exome-sequencing has been done by paired-reads lane 2x100 bp after a SureSelect® array-capture. Both sequencing has been performed on high-throughput Illumina HiSeq sequencer. Burrows-Wheeler Aligner (BWA) was used for the mapping. Briefly, SNPs were called from DNA exome-seq data using The Genome Analysis Toolkit (GATK, Broad Institute) and dbSNP database. For allelic expression analysis, we then only kept SNPs supported by >= 10 RNA-seq reads. For each gene, when several SNPs were informative, we assessed the allelic expression from the most informative SNP. We next calculated the allelic expression ratio as (100-(absolute(Allele A%-Allele B%))). Genes above 40 are considered as bi-allelically expressed and genes below 40 are categorized as mono-allelically expressed. We noted that remarkably fewer X-linked genes were retained in SK-BR-3, compared to the other

samples. This is likely due to the large region of LOH on the long arm of the X-chromosome in this cell line. NB: We chose to keep the same threshold (of 40) for all cell lines, knowing that we would presumably underestimate the degree of Xi-gene reactivation for the ZR-75-1 line which is trisomic for the X-chromosome. Indeed, when the threshold is change for ZR-75-1 to account for ploidy, this would have led to just one more gene being included as "cancer-specific" escapee: *ARHGEF9*. We therefore chose to keep the same threshold of 40 for all lines, in order to simplified data presentation without impacting on the general conclusions.

Chromatin Immunoprecipitation analysis

Chromatin immunoprecipitation assays and massive parallel sequencing

Cancer and normal cells were fixed with 1% para-formaldehyde during 30 minutes at room temperature. Chromatin from fixed cells was fragmented by sonication and immunoprecipitated in lysis buffer (50mM TrisHCl pH=8, 1mM EDTA, 140mM NaCl, 1% Triton, 0.1% Na-deoxycholate) complemented with protease inhibitor cocktail (Roche cat# 11873580001). After overnight immunoprecipitation in presence of the corresponding antibodies, 2 washes with lysis buffer, 2 washes with lysis buffer containing 360mM NaCl, 2 washes with washing buffer (10mM TrisHCl pH=8, 250mM LiCl, 0.5% NP-40, 1mM EDTA, 0.5% Na-deoxycholate) and 2 washes with 1xTE were performed before chromatin elution at 65°C (15 min in elution buffer: 50mM TrisHCl pH=8, 10mM EDTA, 1% SDS). The immunoprecipitated chromatin was decrosslinked overnight (65°C in presence of 1%SDS; 1xTE solution), the remaining proteins were removed by proteinase K treatment (Roche; cat# 03115852001) and phenol-chloroform extraction. The purified DNA was validated by quantitative real-time PCR (qPCR, Roche LC480 light cycler device; Qiagen Quantitect PCR reagents) and libraries for massive parallel sequencing were prepared following standard procedures (NEXTflexTM ChIP-seq library kit; cat# 514120). Chromatin immunprecipitation assays were performed with antbodies directed against RNA Polymerase II (Santa Cruz; sc-9001; H-224), H3K4me3 (Abcam; ab8580) and H3K27me3 (Millipore; ab07449).

ChIP-seq libraries were prepared according to the standard Illumina protocol and sequenced with the HiSeq2500 system. Single end sequencing was carried out to obtain around 25-150 million (M), 100bp long reads per sample.

Alignment and Quality control

Datasets were subjected to two types of quality control. FASTQC-0.10.1 (http://www.bioinformatics.babraham.ac.uk/projects/fastqc/) was used to assess the quality of sequencing and potential adapter or cross contaminants. Average sequencing quality (phred score) per base was above 30 ($Q \geq 30$) for all datasets. In addition, aligned datasets were then subjected to NGS-QC ((Mendoza-Parra et al. 2013); www.ngs-

qc.org) to assess the robustness of enrichment. The majority of data sets were of «triple A» quality, no data set was below «triple B».

For exome-seq and ChIP-seq, alignment was performed using BWA-MEM-0.7.7 (Li and Durbin 2009) with default parameters, which simultaneously checks for both global and local alignment for reads. Alignment was followed by three sets of filters to prevent bias in the analysis. 1) duplicate reads (PCR clonal reads) were filtered out using Picard tools-1.86 (http://picard.sourceforge.net) 2) reads with mapping quality less than 10 were filtered out using Bamtools-2.2.3 (https://github.com/pezmaster31/bamtools) and 3) reads with more than one alignment reported were filtered out using in-house scripts. Further analysis was carried out on processed alignment file which is around 10-100M reads after these filters.

ChIP Allele specific analysis

To prepare the allele information for each cell-line for allele-specific analysis down the line, SNP analysis was carried out along with Human SNP Array 6.0 data. To identify novel variation (apart from known SNPs from Human SNP Array 6.0 data), SNP analysis was carried out on all three ChIP-seq, Exome-seq and RNA-seq data individually. ChIP-seq data of different marks (H3K4me3, H3K27me3, RNA Pol II and Input) were merged for each cell line to increase the depth and confidence for variation calling. Variation calling was performed for each cell line separately for ChIP-seq and Exome-seq following the best practice GATK-2.6.5 pipeline by filtering reads with Mapping quality ≥ 1 (Van der Auwera GA et al., 2013; http://www.broadinstitute.org/gatk/guide/best-practices?bpm=DNAseq). Variation calling for RNA-seq was carried out following the methods of Piskol, Robert et al., 2013 to avoid artifacts specific to RNA-seq data (Piskol et al. 2013). A final list of allele information was generated by combining the SNP information from the different data sets for each cell line. To increase the allele-specific sensitivity for the alignment, reads were additionally realigned in an allele-specific manner following the method of Satya et al. (Satya et al. 2012). Read counts for each allele and SNP position were extracted for each mark using in-house scripts. SNP positions with at least three reads from both alleles were considered as heterozygous positions.

Peak calling and annotation

Peak calling was performed using HOMER ((Heinz et al. 2010); http://homer.salk.edu/homer/index.html) with default parameters. For H3K27me3 and RNA Pol II, the 'style' parameter was chosen as 'histone' due to the broad patterns for this mark, whereas for H3K4me3, which generally give sharp peaks, the parameter 'factor' was chosen. Genomic context annotation over identified peaks were carried out using the HOMER annotation module but with basic annotation by excluding references other than coding genes and non-coding RNA.

Integration of annotations

A gene-based analysis of annotation integration was carried out using in-house scripts to integrate all annotation from peak and variation calling (informative SNP counts, read depths, homo/heterozygous SNP count and weighted allelic imbalance). To include annotations from regulatory regions 1Kb sequences upstream from the TSS and downstream of the TES were considered. A weighted arithmetic average was calculated for each gene by calculating average Allelic Imbalance (AI) where each SNP's AI was weighted by its read depth.

Normalization of ChIP-seq data

To illustrate the comparisons across cell lines, ChIP-seq data were normalized using an in-house developed tool called 'Epimetheus', which is based on quantile normalization (manuscript under preparation). Read Count Intensity (RCI) was calculated for a window of 100bp bin size across chromosomes and then these intensities were normalized using quantile normalization from the limma package. The impact of normalization was assessed using MA plots before and after normalization. Specified genomic feature based normalized RCI was constructed, which are illustrated in Figure 6. For TSS-centered plots and heatmaps, a separate TSS-based normalization was carried out with 20bp bin size to obtain higher resolution.

RNA, DNA FISH and immunofluorescence.

For DNA FISH, cells were denatured in 50% formamide/2X SSC at 80°C for 30 min and rinsed several times in cold 2X SSC prior to overnight hybridization at 42°C. Labeled BAC probes were denatured and competed with Cot-1 DNA (3 µg/coverslip) for 15 min at 37°C. Preparation of the X chromosome paint probe was performed according to the supplier's instructions (CytoCell). After hybridization, coverslips were washed three times in 50%formamide/ 2X SSC and three times in 2X SSC at 42°C for RNA-FISH and DNA-FISH, and then stained with DAPI (0.2 mg/ml). Immunofluorescence RNA-FISH was performed as described previously (Chaumeil et al. 2008). For immunofluorescence, the following antibodies were used: RNA polymerase II (clone CTD 4H8; Millipore cat# 05-623), H3K9Ac (Millipore cat# 06-942), H4Ac (Millipore cat# 06-946), H3K27me3 on interphase (clone 7B11), H3K27me3 on metaphase (ActiveMotif cat# 39155), H3K4me2 (Millipore cat# 07-030) at a 1/200 dilution. For RNA FISH on tumors, either tumor stamps were generated from fresh tissue samples and immediately frozen at -80°C; or else cryosections (10µm thick) were generated. Just prior to IF / FISH, these stamps/sections were fixed in 3% paraformaldehyde/PBS for 10 min, then permeabilized in 1X PBS/0.5% Triton X-100/2 mmol/L vanadyl ribonucleoside complex (New England Biolabs) on ice for 4 min. After three washes in PBS, the sample was

dehydrated through an ethanol series of washes prior to RNA FISH or DNA FISH, which was then performed as described previously (Vincent-Salomon et al. 2007). For immunofluorescence alone, samples were used directly, without prior ethanol treatment and dehydration.

Sequential Immunofluorescence and RNA FISH analysis with super resolution OMX® microscopy

Sequential Immuno-RNA FISH was performed as previously described (Chaumeil et al. 2008). Antibodies used for immuno-staining were: anti-H3K9Ac (Millipore cat# 06-942), anti-H3K27me3 (clone 7B11), anti-H3K4me2 (Millipore cat# 07-030) and anti-RNA polymerase II (clone CTD 4H8; Millipore cat# 05-623). Structured illumination image acquisition was carried out using a DeltaVision OMX version 3 system (Applied Precision, Issaquah, WA) coupled to three EMMCD Evolve cameras (Photometrics, Tucson, AZ). Multi-channel image alignment was performed using ImageJ (Rasband, W.S., ImageJ, U. S. National Institutes of Health, Bethesda, Maryland, USA, http://imagej.nih.gov/ij/, 1997-2012) and UnwarpJ plugin (Sorzano et al. 2005). At least, thirteen nuclei were analyzed for each experiment.

Data processing of 104 Basal-like breast carcinomas (BLC)

We obtained controlled access to the EGA datasets from the study EGAS00001000132 including Human SNP Array 6.0-arrays, RNA exome and whole genome sequencing data (Shah et al. 2012). Human SNP Array 6.0-arrays were processed using the GAP method to obtain absolute copy number and allelic content profiles (Popova et al. 2009). Samples that were classified as bad, average quality or contaminated by normal tissue were discarded. In details, we obtained 81 primary BLC tumors with RNA-seq and SNP-array data available. 39 samples classified as “bad” quality were removed from the analysis: 2 cases were identified as “normal”; 7 cases were identified with bad quality hybridization to SNP-arrays and 30 cases were identified with more than 50% contamination by the normal tissue, which showed low signal to noise ratio in SNP-array copy number and allelic imbalance profiles. Finally, 42 BLCs with good SNP-array quality (i.e. <50% contamination by the normal tissue and high signal to noise ratio) and RNA-seq data available were analyzed (cf Table S2). After evaluation of allelic status of X chromosome, we ended up with 25 samples exhibiting heterozygosity, of at least some region of the X chromosome for further evaluation. Allelic

expression was obtained as the number of reads and corresponding allelic frequency covering known SNP positions. SNPs coverage was obtained based on the SAMtools pileup processing of RNA-seq data (Li et al. 2009).

Supplemental Figure Legends

Figure S1. Xi characterization in normal and cancer cells

(A) Whole X chromosome DNA FISH (X paint in white). Continue of the Figure 1A.

(B) *XIST* expression level assessed by real-time PCR. Normalization was performed using *TBP* expression levels (Kwon et al. 2009). Data represent the mean values +/- SEM.

(C) *XIST* RNA FISH signal intensity. Quantification was performed using ImageJ software (NIH, Betesda) by quantifying FISH signal at *XIST* RNA domain (we normalized *XIST* intensity signal to the general RNA FISH background that we measured in proximity to the Xa identify by using HDAC8 RNA FISH). The number of nuclei analyzed is indicated at the bottom of the box plot.

(D) DAPI signal intensity. Quantification was performed using ImageJ software by comparing the DAPI signal at the *XIST* RNA domain versus DAPI signal associated with the *HDAC8* RNA FISH signal at the Xa. The number of nuclei analyzed is indicated at the bottom of the box plot.

(E) Example of DAPI signal intensity quantification on HMECs and ZR-75-1 cells.

(F) X-paint DNA FISH on metaphase spreads (grey) shows the number of X chromosomes (red) in normal and breast cancer cell lines.

(G) *KDM5C* and *HUWE1* expression at *XIST* domain assessed by nascent transcript RNA FISH (*KDM5C*, red; *HUWE1*, green; *XIST, grey)*

Left Bar chart: Quantification (in %) of nuclei showing mono- or bi-allelic expression of *HUWE1* associated with *XIST* RNA domain.

Right Bar chart: Quantification (in %) of nuclei showing mono- or bi-allelic expression of *KDM5C* associated with *XIST* RNA domain.

(H) Boxplot of the relative levels of *XIST* RNA coating and Cot-1 RNA exclusion. The number of nuclei analyzed is indicated at the bottom of the box plot. Examples of nuclei used for this analysis are shown in figures 1C. The quantification has been done by ImageJ software on images acquired on a Nikon confocal spinning disk microscope. For details on quantification method see Figure S2A and S2C.

(I) Visual quantification of presence or exclusion of Cot-1 RNA at *XIST* RNA domain. The number of nuclei analyzed is indicated at the bottom of the bar chart. Quantification has been done on images acquired on a Nikon confocal spinning disk microscope.

(J) Boxplot of the relative levels of *XIST* RNA coating and RNA Pol II exclusion. The number of nuclei analyzed is indicated at the bottom of the box plot. Examples of nuclei used for this analysis are shown in figures 1C. The quantification has been done by ImageJ software on images acquired on a Nikon confocal spinning disk microscope. For details on quantification method see figure S2A and S2C.

(K) Visual quantification of RNA Pol II presence or exclusion at *XIST* RNA domain. The number of nuclei analyzed is indicated at the bottom of the bar chart. Quantification has been done on images acquired on a Nikon confocal spinning disk microscope.

Box plot on this figure: Upper whisker represents 90%, upper quartile 75%, median 50%, lower quartile 25% and lower whisker 10% of the dataset for each cell line.

(***) $p < 0.001$, (**) $p<0.01$, (*) $p<0.05$ using the Student's *t*-test. All the dataset are compared with HMEC dataset.

Scale bar: 5µm

Figure S2. Histone post-translational modifications associated with the X chromosome

(A) Schematic view of the quantification done by ImageJ home-made macro to evaluate the enrichment / depletion of immuno-staining (or RNA FISH) signal at *XIST* domain compare to the non-*XIST* coated DNA. This quantification has been carried-out on immuno-RNA FISH images (see Figure 1C for example). Nucleoli have not been considered in the analysis. For MDA-MB-436 cells, to avoid bias in the quantification, we also excluded from the analysis the highly H3K27me3 enriched bodies which do not belong to X chromosome (nor in metaphase (Figure 3C) or in interphase (Figure S3F)). NB: Evaluation of the H3K27me3 enrichment on the inactive X chromosome was also performed using an X-chromosome DNA FISH paint (Figure S3F) to identify the Xi territory. This revealed the same changes in H3K27me3 enrichment in cancer cell lines as when *XIST* was used as a read out for the Xi.

(B) Example of results obtained with ImageJ macro in the evaluation of H3K27me3 enrichment at *XIST* domain.

(C) Example of results obtained with ImageJ macro in the evaluation of Cot-1 RNA depletion at *XIST* domain.

(D) Pearson's co-localization coefficients have been evaluated for *XIST* RNA coating and H3K27me3 association on DeltaVision OMX microscope. Examples of nuclei used for the analysis are shown in Figures 2C. Data represent the mean values +/- SEM. The number of nuclei analyzed is indicated at the bottom of the box plot.

(E) Immuno-RNA FISH revealing the degree of H4ac (green) depletion at *XIST* RNA domains (red).

(F) Boxplot of the relative levels of *XIST* RNA coating and H4ac association. The number of nuclei analyzed is indicated at the bottom of the box plot. The quantification has been done by ImageJ software on images acquired on a Nikon confocal spinning disk microscope. For details on quantification method see Figure S2A and S2C.

(G) Immuno-RNA FISH revealing the degree of H3K4me2 (green) depletion at *XIST* RNA domains (red).

(H) Boxplot of the relative levels of *XIST* RNA coating and H3K4me2 association. The number of nuclei analyzed is indicated at the bottom of the box plot. The quantification has been done by ImageJ software on images acquired on a Nikon confocal spinning disk microscope. For details on quantification method see Figure S2A and S2C. Similar results have been obtained by immuno-RNA FISH for H3K4me3 and *XIST* RNA (Data not shown).

(I) Visual quantification at *XIST* RNA coating of H3K27me3 enrichment; H3K9ac exclusion; H3K4me2 exclusion and H4ac exclusion. The number of nuclei analyzed is indicated at the bottom of the bar chart. Examples of nuclei used for this analysis are shown in figures 2A, 2D, S2E and S2G. Quantification has been done on images acquired with Nikon confocal spinning disk microscope.

(J) Immuno-RNA-FISH for *XIST* (red) and H3K4me2 (green). Acquisition has been carried out by super-resolution structured illumination on DeltaVision OMX microscope. Inset for H3K4me2, *XIST* RNA and merge is shown below each cell lines.

(K) Immuno-RNA FISH for *XIST* (red) and RNA Pol II (green). Acquisition has been carried out by super-resolution structured illumination on DeltaVision OMX microscope. Inset for RNA Pol II, *XIST* RNA and merge is shown below each cell lines.

(***) $p < 0.001$, (**) $p<0.01$, (*) $p<0.05$ using the Student's *t*-test. All the dataset are compared with HMEC dataset.

Box plot on this figure: Upper whisker represents 90%, upper quartile 75%, median 50%, lower quartile 25% and lower whisker 10% of the dataset for each cell line.

Figure S3. Nuclear organization of the *XIST* RNA coated chromosome

(A) Genomic DNA Sanger sequencing of *XIST* provides genotype information for at least one SNP in each tumoral cell lines. cDNA sequencing reveals that *XIST* is mono-allelically expressed in the three tumor cell lines.

(B) Bar chart of the percentage of nuclei observed as EdU positive or EdU negative. Briefly, we performed a 30min EdU pulse on cultured cells and then detected incorporated Edu using the Click-It assay (Invitrogen). At least 100 nuclei were analyzed for each cell line. Indeed, we wondered whether the perturbed state of the Xi in some cells might be dependent on cell cycle, for example due to S phase when chromatin must be replicated. Thus, we further examined MDA-MB-436 cells, which showed the highest proliferation rate of the three cancer cell lines.

(C) The MDA-MB-436 cell line was used for a sequential IF / RNA FISH (EdU pulse and detection / H3K27me3 immuno-staining / *XIST* RNA FISH). Using ImageJ macro, we quantified the degree of H3K27me3 enrichment in EdU positive and EdU negative cells to explore the impact of cell cycle on Xi epigenetic chromatin mark instability in tumoral cell lines. The level of H3K27me3 enrichment is slightly lower in this experiment compare to Figure 2B, presumably due to a slight decrease in immuno-staining quality following EdU “Click-It” detection. No particular correlation could be seen between EdU positive (S phase) or EdU negative (G1 or G2 phase) cells and disrupted H3K27me3 enrichment on the Xi , indicating that the disrupted chromatin patterns observed are not necessarily linked to a specific stage of the cell cycle such as S phase.

(D) Example of H3K27me3 signal intensity quantification on EdU negative or EdU positive MDA-MB-436 cells (Immuno-RNA FISH: DNA, blue; *XIST* RNA, red; EdU, green and H3K27me3, white).

(E) Immuno-blotting was performed on protein nuclear extracts prepared as follows. After washing with PBS, cells were incubated on ice for 10 minutes in buffer A (10 mM HEPES pH 7.8, 10 mM KCl, 2 mM MgCl2, 0.1 mM EDTA) with protease inhibitor cocktail, added 10% NP40, and centrifuged at 14,000 rpm for 20 seconds. The supernatant was removed. The pellet was suspended in buffer B (50 mM HEPES pH 7.8, 50 mM KCl, 300 mM NaCl, 0.1 mM EDTA, protease inhibitor cocktail) and incubated on ice for 30 minutes. Nuclear debris was pelleted by centrifugation at 14,000 rpm for 10 minutes, and the supernatants were used as nuclear extracts. Nuclear proteins (20µg) were separated on SDS–polyacrylamide gel electrophoresis and transferred to polyvinylidene difluoride (PVDF) membranes by a standard procedure. Antibodies used for immunoblotting were: H3K27me3 (ActiveMotif cat# 39155), H3K4me2 (Millipore cat# 07-030), H4ac (Millipore cat# 06-946), H3ac (Millipore, cat# 06-599), H3 (Abcam, cat# 1791), Lamin A/C (Millipore, cat# 05-714). Immunoblots were revealed using enhanced chemiluminescence (ECL+, Amersham). Histone 3 and Lamin A/C are used as loading normalization.

(F) Sequential immuno-RNA/DNA FISH was performed as described (Chaumeil et al. 2008). Briefly, staining and images acquisition were first carried out and positions saved. Slides were then treated with RNase A and RNase H for 1h at 37°C. After several washes, slides were used for X chromosome paint DNA FISH. At least, 80 nuclei were analyzed for each cell lines. Grey and red drawing outlines of *XIST* RNA, H3K27me3 and H3K4me2 panels represent the X chromosome territories (from the X chromosome panel). For each cell lines either one or two planes are shown.

Figure S4. Validation of X-linked genes allelic expression status

(A) Schematic outline of our allele-specific transcriptional analysis of X-chromosome transcriptional activity.

(B) Dilution- limited cultures enabled us to derive 22 independent clones from the primary WI-38 cell line. Analysis of each clone by allele-specific PCR reveals a clear mono-allelic expression of *CLCN4* from either the maternal or paternal X chromosome (eleven from each origin were obtained). Amongst this 22 clones, we then chose two clones, displaying inactivation of one or the other X, for RNA-seq and two further clones (again with inactivation of opposite alleles) for the RNA SNP6 approach. Similarly, we also derived clones from HMEC cells by dilution-limit culture. However the cloning efficiency of HMEC cells was far lower than for WI-38 cells. Only two HMEC clones were obtained, both with very limited cell proliferation capacity and were therefore not used for the RNA SNP6 array or RNA-seq analysis but only for allele-specific PCR. Each clone shows a clear mono-allelic expression of *NXT2* from maternal or paternal chromosome (data not shown).

(C-E) We also derived single cell clones from the ZR-75-1, SK-BR-3 and MDA-MB-436 cells. Each tumor cell line revealed the same Xi/Xa allelic profile in all clones analyzed and the parental bulk cell line, as expected if these tumors (and the cell lines derived from them) were originally clonal, unlike WI-38 and HMEC primary cells which are polyclonal, with a mixed population of cells harboring a maternal or paternal inactivated X chromosome. For example, *NXT2* allelic expression is strictly the same between the bulk population and the clones for ZR-75-1 (mono-allelic expression) and MDA-MB-436 (bi-allelic expression).

(F) For the allele-specific transcriptome experiment we used two different WI-38 clones (with alternative paternal Xi / maternal Xi profiles). The allele-specific expression profile obtained on autosomal genes are almost the same in both clones. This demonstrates the robustness and accuracy of the approach and the fact that both clones present a similar allelic expression pattern on autosomes.

(G) WI-38 clone #1 harbors an inactive X chromosome of different parental origin compared to clone #28, however the allelic expression patterns observed were very similar between the two clones revealing that at this level of resolution

there are no striking differences in X-chromosome inactivation status between the maternal and paternal X chromosomes.

(H-I) Allele-specific PCR using TaqMan® probes for the analysis of *HDAC8* rs5912136 (H) and *APOOL* rs4828121 (I). The x axis show expression from allele A and the y axis expression from allele B. RT minus sample was used as negative control (i.e. no amplification of alleles A and B). To determine threshold for 100% of allele A or 100% allele B, we used pure gDNA material. For example, qRT-PCR with TaqMan® probes demonstrated that *APOOL,* is mono-allelically expressed in WI-38 cells, but is bi-allelically expressed in tumor cell lines with similar expression levels from inactive and active alleles in MDA-MB-436 cells, and lower levels (about 30%) from the inactive allele in ZR-75-1 cells.

(J) Genomic DNA Sanger sequencing of several genes provides genotype information for at least one SNP. cDNA sequencing reveals whether the gene expression is mono- or bi-allelic. For example, cDNA and gDNA Sanger sequencing on *SYTL4* reveal an mono-allelic expression in normal WI-38 cells and in the ZR-75-1 cell line, but an bi-allelic expression in MDA-MB-436 cells, (and uninformative in SK-BR-3 cells due to LOH).

Figure S5. Transcriptional activity of the X chromosome

(A) Allele specific PCR based on TaqMan® probes for analysis of *NXT2* rs3204027. The x axis shows expression from allele A and y axis expression from allele B. RT minus sample was used as negative control (i.e. no amplification of alleles A and B). To determine threshold for 100% of allele A or 100% allele B, we used pure gDNA material.

(B) Cancer Testis (C/T) antigen mRNA expression analysis. We investigated expression of several X-linked members of the C/T antigen family. This was performed by normalizing data to *TBP* expression for each sample and then reported to HMEC expression to evaluate expression increased in breast cancer cell lines. These genes are normally only expressed in the testis and are silent in somatic tissues, on both the active and inactive X chromosomes, but have been reported to be over-expressed in breast tumors. We found that some of these genes showed no expression at all (*MAGEA12, SAGE1, XAGE3*, data not shown) in all cell lines examined, while others (*MAGEA4, MAGEA6* and *MAGEC2*) showed increase expression in the cancer cell lines but not in normal cells. The aberrant expression of X-linked C/T antigens could either be due to reactivation on the Xi or the Xa. In the case of SK-BR-3, only the active X chromosome alleles, of those three genes, are present (due to LOH) meaning that the over-expression we observed must be due to the re-activation of the alleles on the active X chromosome. To detect *MAGEA6* expression in the other cell lines, RNA FISH was used. Data represent the mean values +/- SEM.

(C) *MAGEA6* expression analysis by RNA FISH on normal and tumoral cell lines (green). HDAC8 (red) and *XIST* (grey) RNA FISH has been used as control to localize Xi and Xa region within the nucleus. In normal cells (HMEC and

WI-38), no expression of *MAGEA6* was found. *MAGEA6* expression could be detected from the active X in a significant proportion of SK-BR-3 (47%) and ZR-75-1 (32%) cells, but never from the Xi. In MDA-MB-436 cells, as the *MAGEA6* loci are associated with an X chromosome fragment that is no longer linked to the XIC, it was not possible to determine from which allele the gene was expressed.

(D) Z-projections of 3D RNA FISH show representative examples of HDAC8 expression (green) at XIST domains (grey) in normal (WI-38 and HMEC) and breast cancer cell lines (ZR-75-1, SK-BR-3, and MDA-MB-436). In SK-BR-3 cells, arrowheads indicate active X chromosomes and arrows XIST-coated chromosomes. On the right, bar graph shows levels of HDAC8 expression from XIST domains, with reactivation in SK-BR-3 and MDA-MB-436 cells.

(E) *ATRX* expression assessed by nascent transcript RNA FISH on DAPI-stained nuclei (ATRX, green; *XIST*, grey; DNA, blue).

(F) *TBL1X* expression level assessed by real-time PCR. Normalization was performed using *TBP* expression level. Data represent the mean values +/- SEM.

(***) p< 0.001, (**) p<0.01, (*) p<0.05 using the Student's *t*-test. All the dataset are compared with HMEC dataset.

Figure S6. Perturbation of the Xi chromatin landscape in breast cancer cells

(A) Gene promoter DNA methylation analysis. Each histogram indicates the ratio of promoter methylation (0 to 1) according to gene and to cell line. The position of the gene is indicated on the X chromosome. Color code indicates the known allelic expression status on the Xi for each gene in different cell lines (subject to XCI, blue; escape from XCI, red; LOH i.e. no locus on Xi, brown; unknown; black). Data represent the mean values +/- SEM. Primers used for analysis by EpiTYPER are available in Table S3. DNA methylation levels of X-linked promoters examined were consistent with the Xa:Xi chromosome ratios in different cell lines. For example, in ZR-75-1 a general reduction in DNA methylation of X-linked gene promoters was found compared to HMECs, consistent with the presence of two Xa versus one Xi. This was most pronounced for regions presenting LOH, where only the Xa allele is present.

(B) Variation of H3K27me3 signals of 1Mb bins along the chromosome 17 between HMEC and either Wi-38 or the three tumor cell lines. Above 0 mean more enrichment in HMEC cells, and below 0 more enrichment in the cell line used in the comparison. The profile appears much more variable in the three tumor cell lines than by comparing HMEC to WI-38.

(C) UCSC Genome Browser (Kent et al. 2002) whole X chromosome view of H3K4me3 and H3K27me3 ChIP-seq data. Our data have been normalized (see Materials and Methods for more details). The quality of ChIP-seq data sets was validated with the NGS-QC Generator and received QC Stamps between “triple A” and “BAA” ((Mendoza-Parra et al. 2013); www.ngs-qc.org). In addition to our own HMEC data, HMEC profiles for H3K4me3, H3K27me3 and H3K9me3 were obtained from ENCyclopedia Of DNA Elements (ENCODE) project and were used to : 1- compare the ChIP-seq quality of our dataset; 2- refine the position of the two distinct chromatin type identified on the Xi in normal human cells : H3K9me3 (green) or H3K27me3 (red) enriched. At the bottom, the X chromosome schematic view highlights the H3K9me3 (green) or H3K27me3 (red) enriched domain. The percentages correspond to the frequency of detection of those particular regions in human cells by immuno-staining on metaphase by Chadwick, B (Chadwick 2007). Asterisk indicates a preferentially H3K9me3 enriched region which has not been observed by immuno-staining on metaphase by Chadwick, B, but which is clearly visible by ChIP-seq analysis on HMEC likely due to the higher resolution.

(D) TSS-centered plots for RNA Pol II and H3K4me3 enrichment of X-linked subject to XCI or escaping from the XCI. For each cell lines, X-linked subject to the XCI or escaping from the XCI have been choose based on RNA-seq and RNA SNP6 analyzed done previously (cf Table S1) (but for *HMEC, as we do not have allelic expression analysis available, we used X-linked genes list obtained from analyzing WI-38 clones). Genes escaping the XCI show higher enrichment of RNA Pol II and H3K4me3 at the TSS region, indicating that expression of an additional copy is sufficient to observe enrichment increase. Furthermore, in ZR-75-1 cells, there two active X and one inactive X chromosome meaning that we are still detecting expression of one additional copy out of three (even though the difference is reduced compare to the other cell lines).

(E) Heatmap for enrichment of RNA Pol II and H3K4me3 at the TSS region. We represented heights X-linked: four are escaping XCI in all the cell lines (*KDM6A*, *KDM5A*, *RPS4X* and *SMC1A*) and four are silenced on the Xi in all the cell lines (*RBM41*, *DLG3*, *HUWE1* and *RRAGB*).

Figure S7. Local perturbation of the Xi chromatin in breast cancer cells

(A) TSS-centered plots (+/- 1.5kb) show RNA Pol II and H3K4me3 enrichment for all the X-linked genes (except regional Xi loss) of each tumoral cell lines and HMEC. The number of genes analyzed is indicated below each plot.

(B) Heatmap for RNA Pol II and H3K4me3 enrichment at TSS +/- 1,5 kb. Genes listed are “cancer-specific” escapees (in respect to each of the three tumoral cell lines; see figure 6D to have the averaging of the TSS region enrichment).

(C) Example of H3K27me3, RNA Pol II and H3K4me3 Allelic Imbalance (AI) enrichment for known escaping or silenced X-linked genes in the three tumoral cell lines. AI has been calculated, for a given gene, by the weighted arithmetic mean of all the informative SNPs lying within the gene body (+1kb before TSS and +1kb after the TES). As for RNA-seq analysis, AI < 40 is considered as a mono-allelic enrichment (i.e. < 20% enrichment for the lowest enriched allele) and above AI > 40 as bi-allelic enrichment. X-linked genes escaping the XCI show an enrichment of H3K4me3 and RNA Pol II on the active and inactive allele. At the contrary, H3K27me3 is only detected enriched on one allele. X-linked subject to the XCI show mono-allelic enrichment of H3K27me3, RNA Pol II and H3K4me3.

(D) Allelic imbalance (AI) of H3K27me3, RNA Pol II or H3K4me3 enrichment is shown for “cancer-specific” escapees in MDA-MB-436 cells. AI for a given gene represents the weighted mean of informative SNPs lying within the gene body +/- 1kb. As for RNA-seq analysis, AI < 40 is considered as a mono-allelic enrichment.

Figure S8. Assessment of the epigenetic status of the inactive X chromosome in primary breast tumors

(A-D) Examples of RNA FISH for HDAC8, ATRX, TBL1X nascent transcripts and *XIST* RNA; or immuno-RNA FISH for RNA Pol II and H3K27me3 combined with *XIST* RNA FISH on primary breast tumor samples: Luminal A sub-type IDC (Invasive Ductal Carcinoma) of grade II (T2) (A); HER2 amplified sub-type IDC of grade II (T3) (B); Basal-like sub-type IDC of grade III (T4) (C) and lymph node metastasis coming from patient on panel C (T4meta) (D).

Left panel: HDAC8, *ATRX* and *XIST* expression was assessed by RNA FISH on breast tumor stamps.

Middle panel: TBL1X, *MAGEA6* and *XIST* expression was assessed by RNA FISH on stamps of breast tumor stamps.

Right panel: Immuno-RNA FISH reveals the degree of H3K27me3 enrichment (green) and of RNA Pol II (red) depletion on the *XIST* coated chromosome (gray). Acquisition was carried out by super-resolution structured illumination on DeltaVision OMX microscope for the images displayed on the panel. Quantification of RNA Pol II exclusion and H3K27me3 enrichment at *XIST* domain have been carried-out on images acquired with a Nikon confocal spinning-disk microscope and with the DeltaVision OMX microscope.

(E) DAPI signal intensity of the Barr body. Quantification was performed on primary breast tumor samples and healthy breast tissues using ImageJ software by comparing the DAPI signal at the *XIST* RNA domain versus DAPI signal associated with the ATRX RNA FISH signal at the Xa. The number of nuclei analyzed is indicated at the bottom of the box plot. (***) p< 0.001, (*) p<0.05 using the Student's *t*-test. All the dataset are compared with healthy breast tissue #1.

(F) Example of DAPI signal intensity quantification on one healthy breast tissue and one luminal A sub-type IDC of grade II (T1).

Scale bar : 10µM

Figure S9. Assessment of the inactive X chromosome transcriptional reactivation in primary breast tumors

(A) Description of allelic expression analysis performed with dataset from 104 Basal-like breast tumors (Shah et al. 2012).

(B) The left chart shows allelic expression status of the 183 informative X-linked genes expressed in the 25 selected tumors (see Figure S9A). The right chart indicates allelic expression status of the 78 bi-allelically expressed genes in normal non-tumor cells (Cotton et al. 2013).

(C) List of genes that escape in at least two primary breast tumors in a “cancer-specific” manner. The three genes in red were already identified as escapees in the three tumor cell lines (see Table S1).

Figure S10. Summary of the inactive X epigenetic erosion in breast cancer cells

(A) Summary table of the overall inactive X-chromosome status in normal and breast cancer cells.

(B) Schematic view of the inactive X chromosome erosion in breast cancer cells.

Table S1. List of the genes identified as subject to XCI or to escape from XCI in the WI-38 clones, ZR-75-1, SK-BR-3 and MDA-MB-436.

Table S2. List of the “good quality” primary breast tumors analyzed based on Shah et al dataset (Shah et al. 2012).

Table S3. List of primers used.

Supplementary references

Chadwick BP. 2007. Variation in Xi chromatin organization and correlation of the H3K27me3 chromatin territories to transcribed sequences by microarray analysis. *Chromosoma* **116**(2): 147-157.

Chaumeil J, Augui S, Chow JC, Heard E. 2008. Combined immunofluorescence, RNA fluorescent in situ hybridization, and DNA fluorescent in situ hybridization to study chromatin changes, transcriptional activity, nuclear organization, and X-chromosome inactivation. *Methods Mol Biol* **463**: 297-308.

Cotton AM, Ge B, Light N, Adoue V, Pastinen T, Brown CJ. 2013. Analysis of expressed SNPs identifies variable extents of expression from the human inactive X chromosome. *Genome biology* **14**(11): R122.

Heinz S, Benner C, Spann N, Bertolino E, Lin YC, Laslo P, Cheng JX, Murre C, Singh H, Glass CK. 2010. Simple combinations of lineage-determining transcription factors prime cis-regulatory elements required for macrophage and B cell identities. *Molecular cell* **38**(4): 576-589.

Kent WJ, Sugnet CW, Furey TS, Roskin KM, Pringle TH, Zahler AM, Haussler D. 2002. The human genome browser at UCSC. *Genome research* **12**(6): 996-1006.

Kwon MJ, Oh E, Lee S, Roh MR, Kim SE, Lee Y, Choi YL, In YH, Park T, Koh SS et al. 2009. Identification of novel reference genes using multiplatform expression data and their validation for quantitative gene expression analysis. *PloS one* **4**(7): e6162.

Li H, Durbin R. 2009. Fast and accurate short read alignment with Burrows-Wheeler transform. *Bioinformatics* **25**(14): 1754-1760.

Li H, Handsaker B, Wysoker A, Fennell T, Ruan J, Homer N, Marth G, Abecasis G, Durbin R. 2009. The Sequence Alignment/Map format and SAMtools. *Bioinformatics* **25**(16): 2078-2079.

Mendoza-Parra MA, Van Gool W, Mohamed Saleem MA, Ceschin DG, Gronemeyer H. 2013. A quality control system for profiles obtained by ChIP sequencing. *Nucleic acids research* **41**(21): e196.

Piskol R, Ramaswami G, Li JB. 2013. Reliable identification of genomic variants from RNA-seq data. *American journal of human genetics* **93**(4): 641-651.

Popova T, Manie E, Stoppa-Lyonnet D, Rigaill G, Barillot E, Stern MH. 2009. Genome Alteration Print (GAP): a tool to visualize and mine complex cancer genomic profiles obtained by SNP arrays. *Genome biology* **10**(11): R128.

Satya RV, Zavaljevski N, Reifman J. 2012. A new strategy to reduce allelic bias in RNA-Seq readmapping. *Nucleic acids research* **40**(16): e127.

Shah SP, Roth A, Goya R, Oloumi A, Ha G, Zhao Y, Turashvili G, Ding J, Tse K, Haffari G et al. 2012. The clonal and mutational evolution spectrum of primary triple-negative breast cancers. *Nature* **486**(7403): 395-399.

Sorzano CO, Thevenaz P, Unser M. 2005. Elastic registration of biological images using vector-spline regularization. *IEEE transactions on bio-medical engineering* **52**(4): 652-663.

Vincent-Salomon A, Ganem-Elbaz C, Manie E, Raynal V, Sastre-Garau X, Stoppa-Lyonnet D, Stern MH, Heard E. 2007. X inactive-specific transcript RNA coating and genetic instability of the X chromosome in BRCA1 breast tumors. *Cancer Res* **67**(11): 5134-5140.

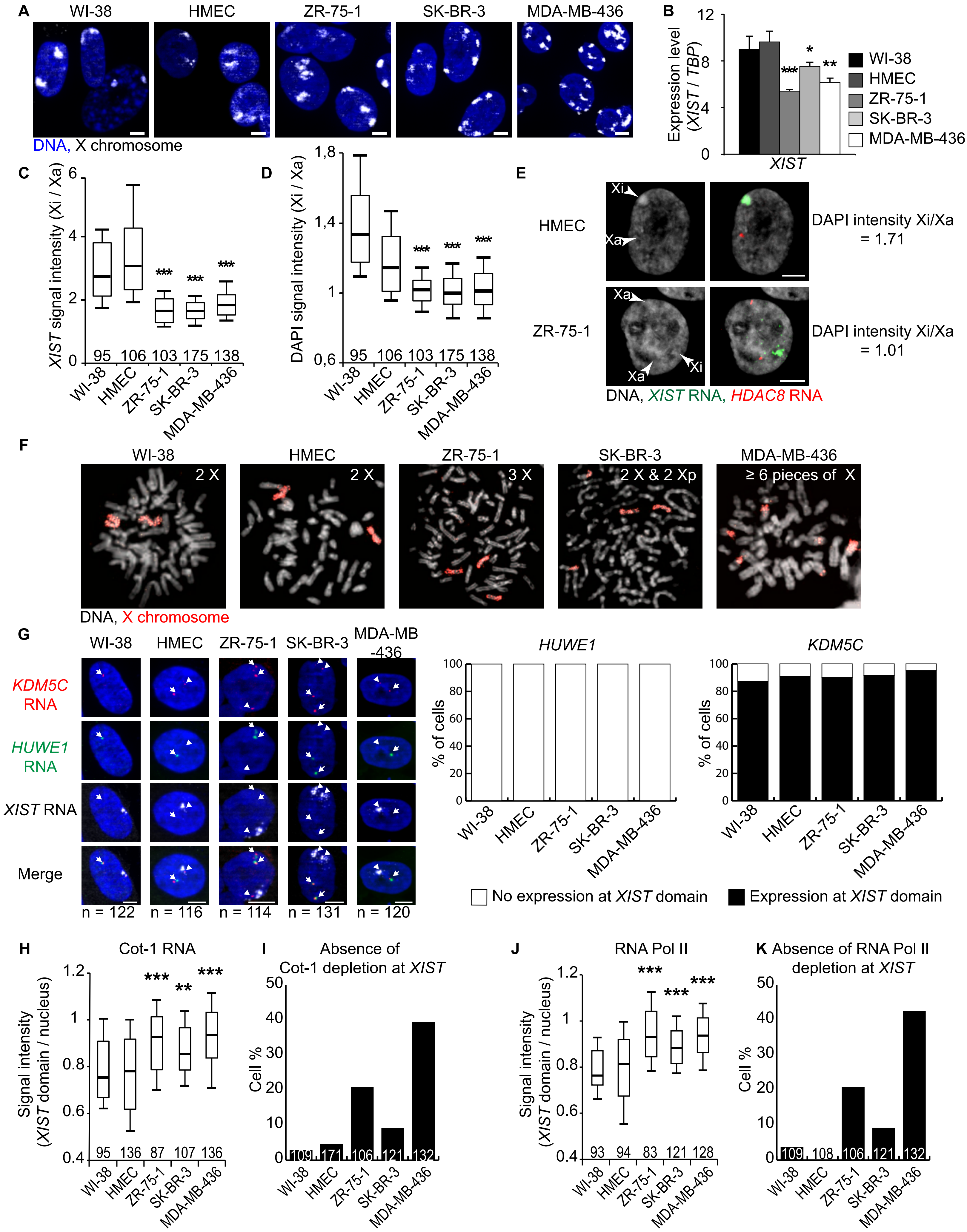

Fig S1, Chaligné et al.

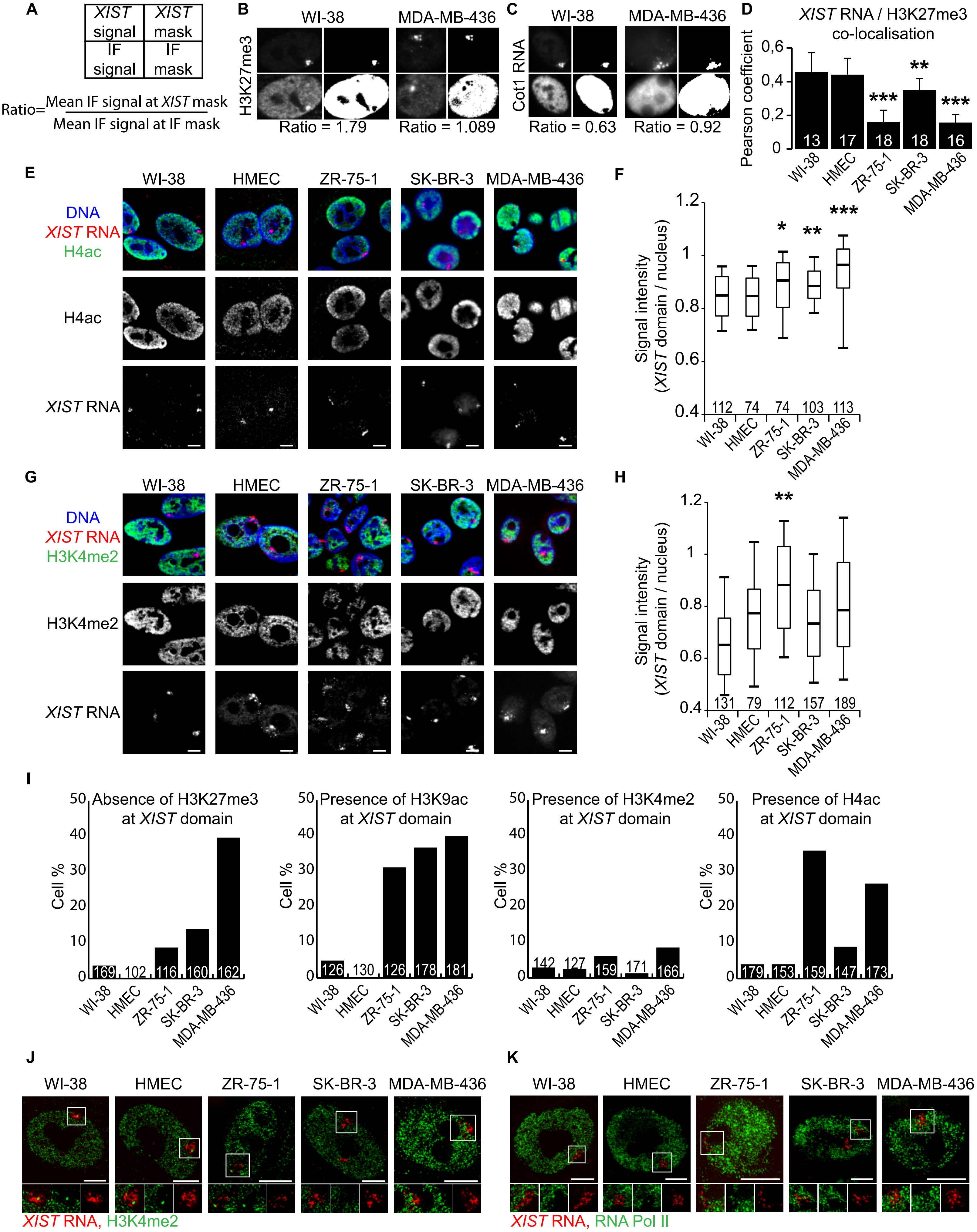

Fig S2, Chaligné et al.

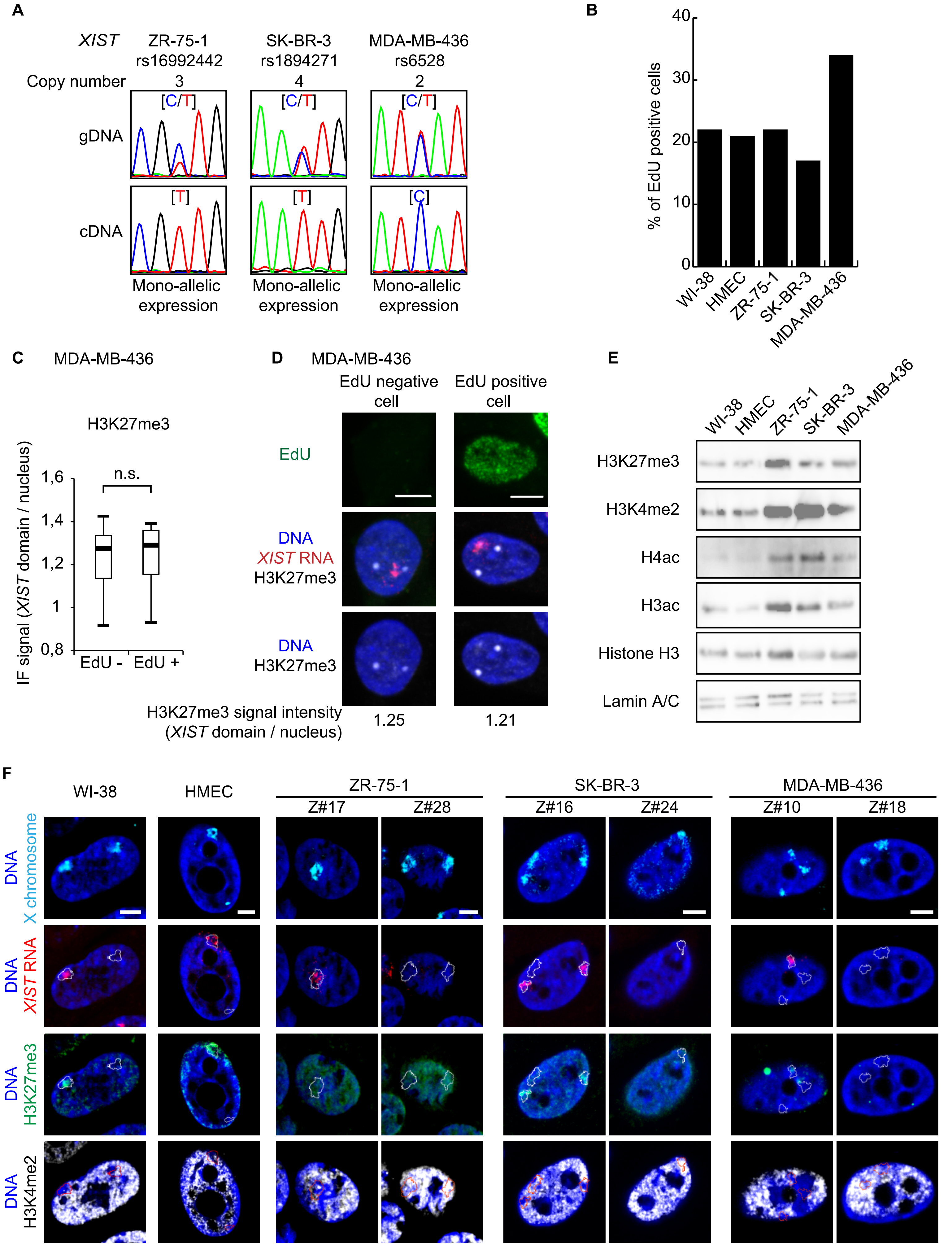

Fig S3, Chaligné et al.

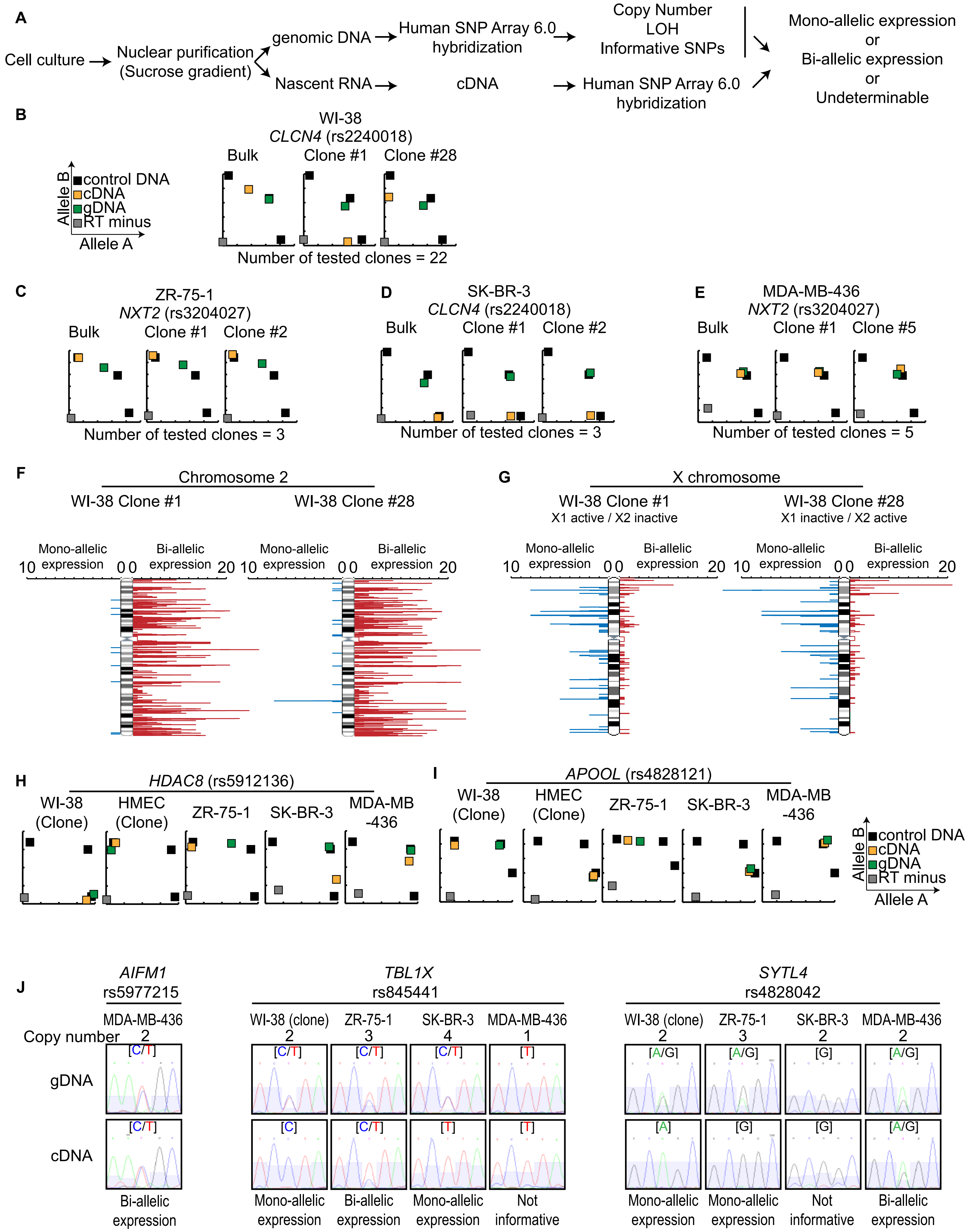

Fig S4, Chaligné et al.

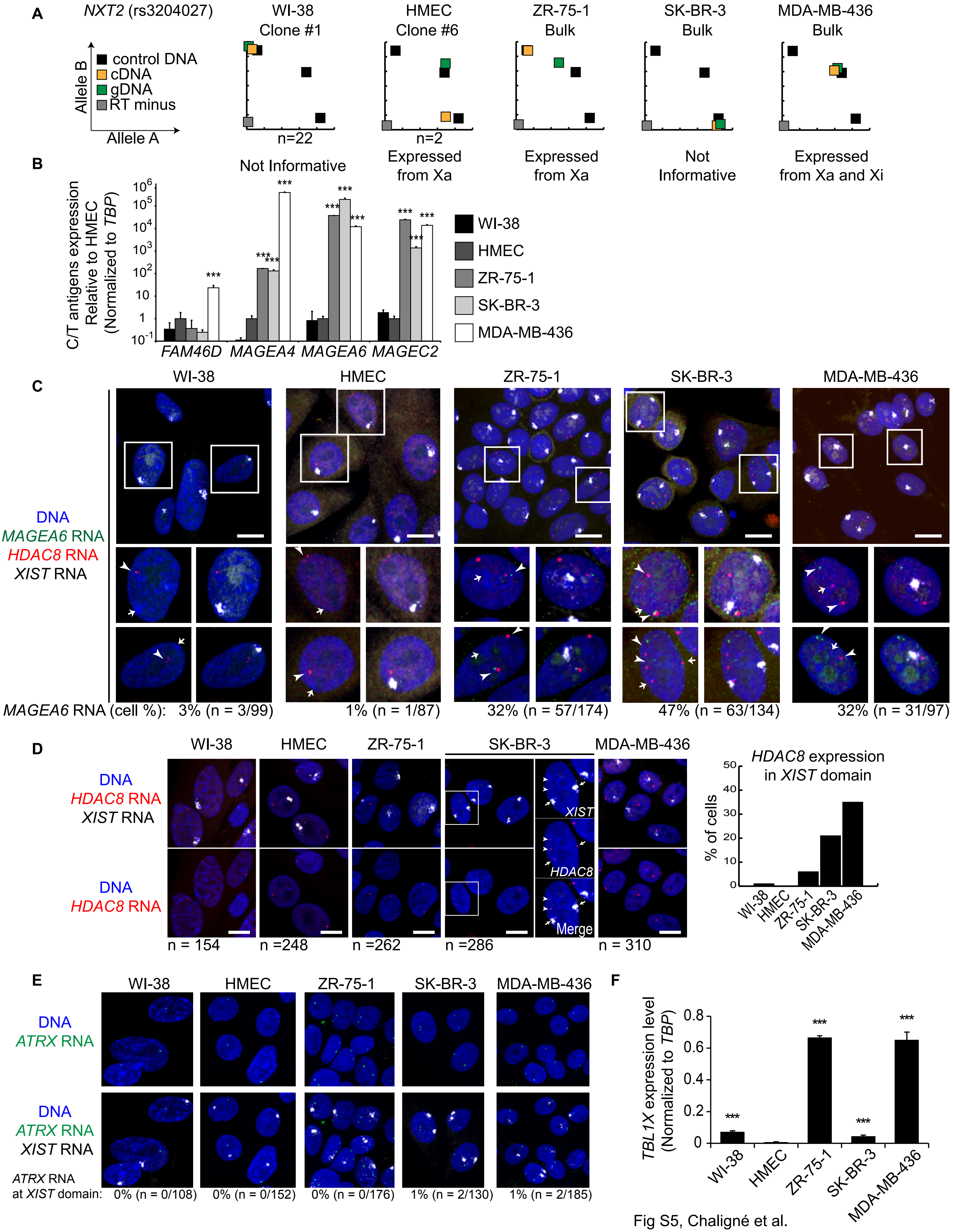

Fig S5, Chaligné et al.

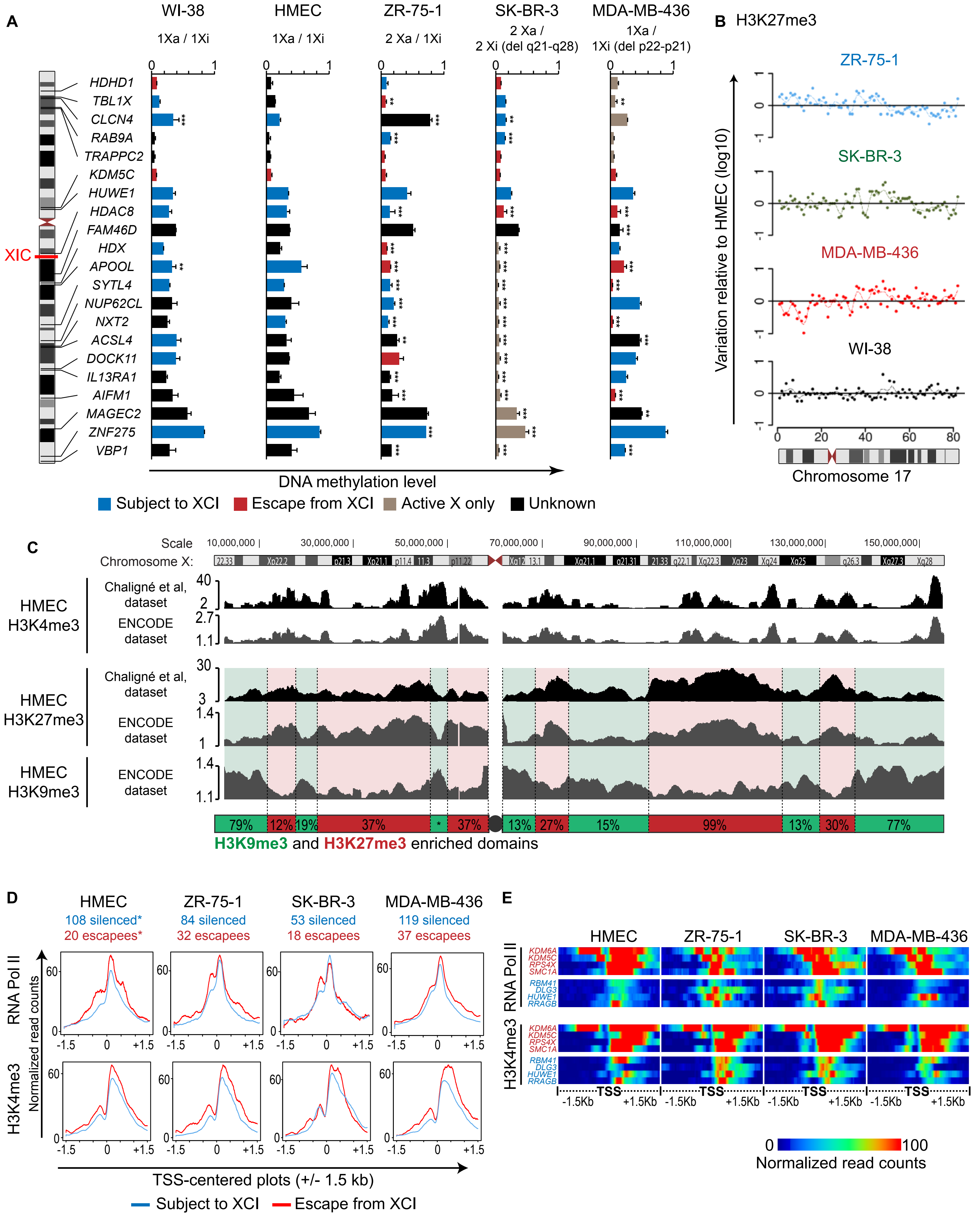

A
WI-38
1Xa / 1Xi
HMEC
1Xa / 1Xi
ZR-75-1
2 Xa / 1Xi
SK-BR-3
2 Xa /
2 Xi (del q21-q28)
MDA-MB-436
1Xa /
1Xi (del p22-p21)
XIC
HDHD1
TBL1X
CLCN4
RAB9A
TRAPPC2
KDM5C
HUWE1
HDAC8
FAM46D
HDX
APOOL
SYTL4
NUP62CL
NXT2
ACSL4
DOCK11
IL13RA1
AIFM1
MAGEC2
ZNF275
VBP1
DNA methylation level
Subject to XCI
Escape from XCI
Active X only
Unknown
B
H3K27me3
ZR-75-1
SK-BR-3
MDA-MB-436
WI-38
Variation relative to HMEC (log10)
Chromosome 17
C
Scale
Chromosome X:
HMEC
H3K4me3
Chaligné et al, dataset
ENCODE dataset
HMEC
H3K27me3
Chaligné et al, dataset
ENCODE dataset
HMEC
H3K9me3
ENCODE dataset
79%
12%
19%
37%
*
37%
13%
27%
15%
99%
13%
30%
77%
H3K9me3 and H3K27me3 enriched domains
D
HMEC
108 silenced*
20 escapees*
ZR-75-1
84 silenced
32 escapees
SK-BR-3
53 silenced
18 escapees
MDA-MB-436
119 silenced
37 escapees
RNA Pol II
H3K4me3
Normalized read counts
TSS-centered plots (+/- 1.5 kb)
Subject to XCI
Escape from XCI
E
HMEC
ZR-75-1
SK-BR-3
MDA-MB-436
RNA Pol II
H3K4me3
KDM6A
KDM5C
RPS4X
SMC1A
RBM41
DLG3
HUWE1
RRAGB
TSS
-1.5Kb
+1.5Kb
Normalized read counts

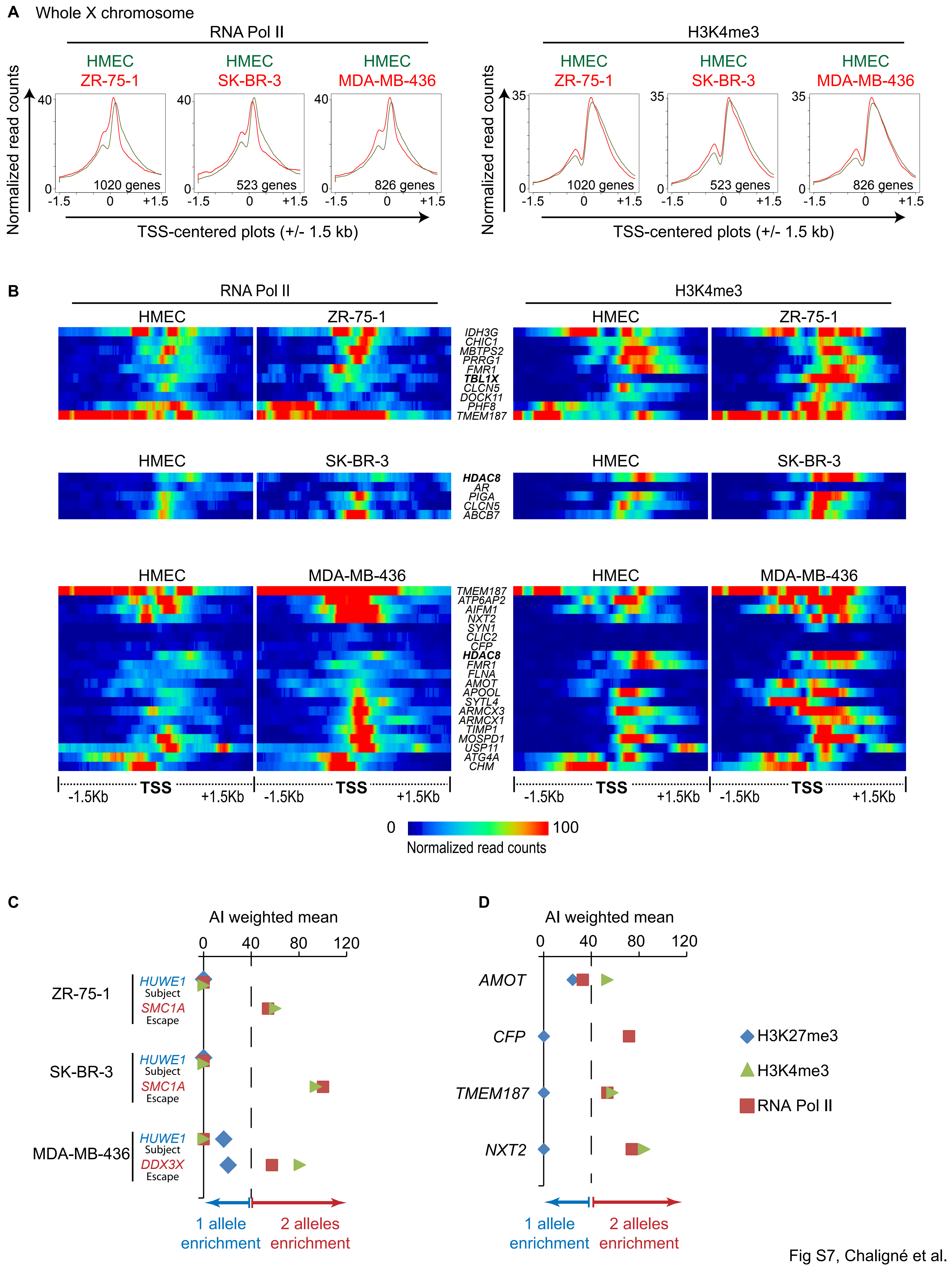

Fig S7, Chaligné et al.

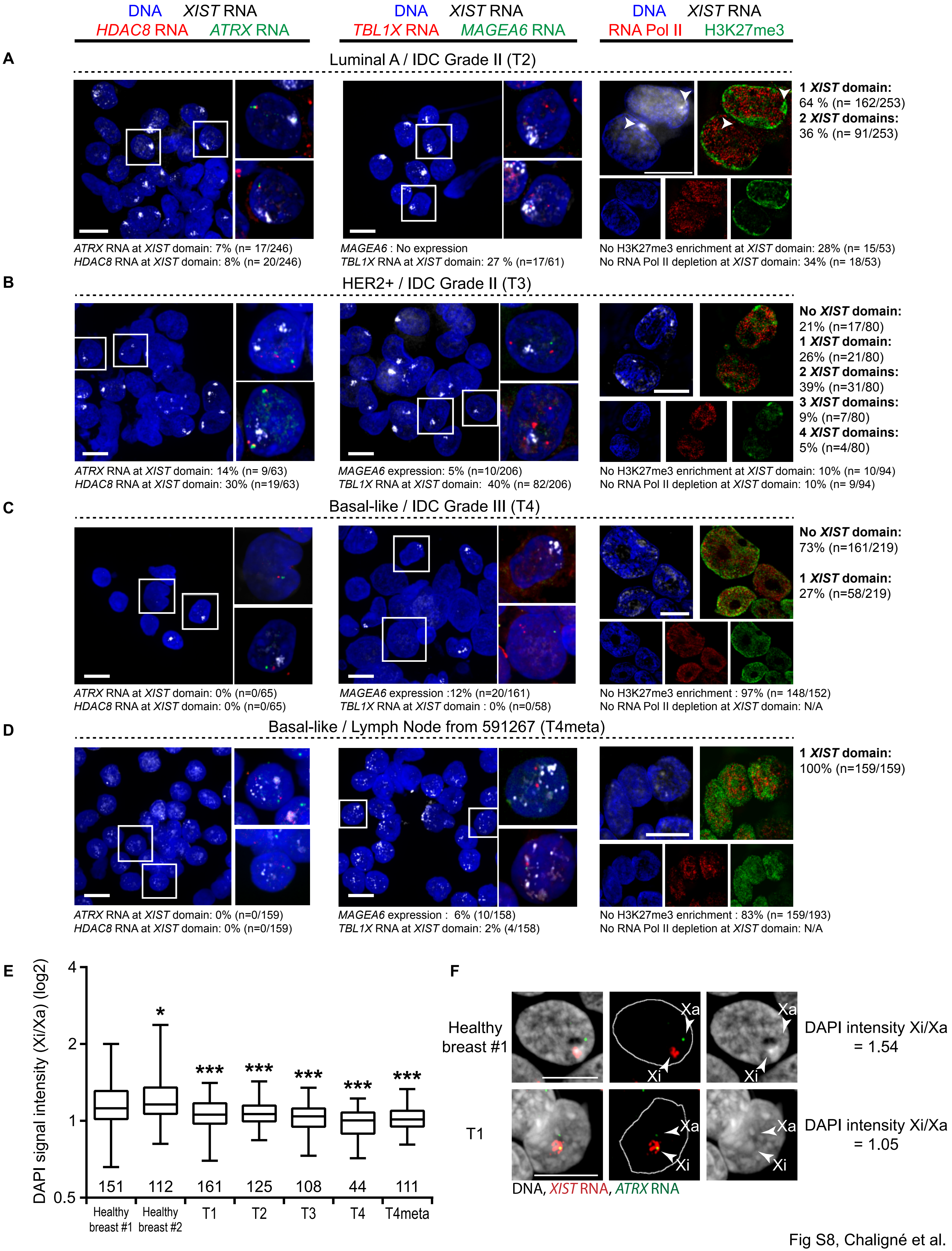

Fig S8, Chaligné et al.

A

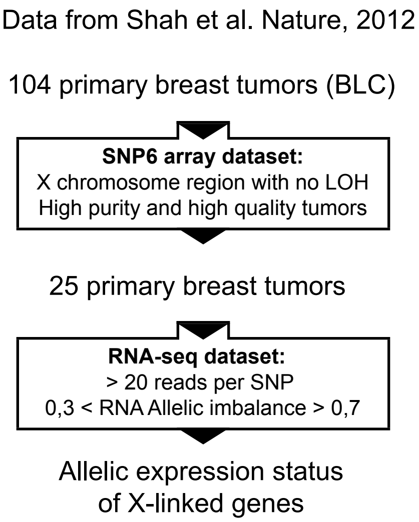

B

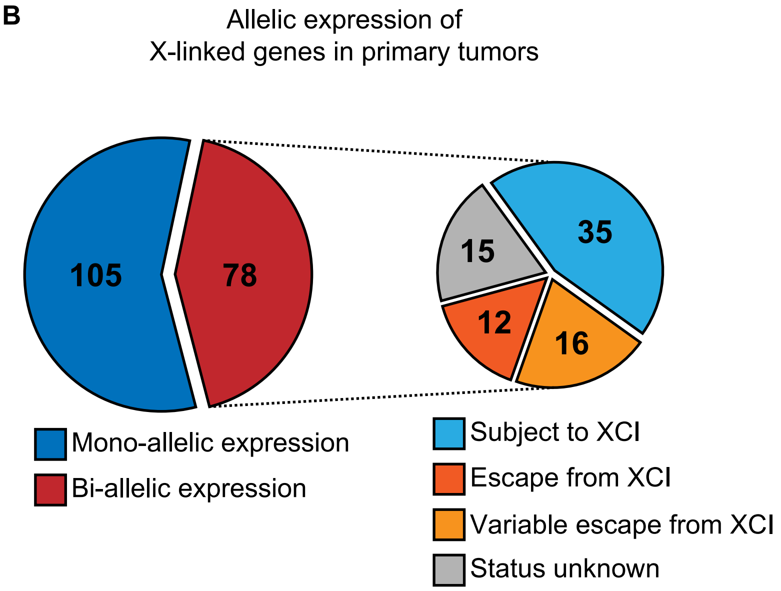

C

Gene	Tumor number: Bi-allelic expression	Tumor number: Mono-allelic expression
GNL3L	14	2
TMEM164	4	1
XIAP	4	9
CYBB	3	1
DOCK11	3	7
NXT2	3	3
TBL1X	3	5
CLCN4	2	6
PJA1	2	8
SASH3	2	8
SH3BGRL	2	3
SLC25A43	2	5
TCEAL4	2	4
ZNF275	2	9

Fig S9, Chaligné et al.

A

	Xi genetic pattern	Barr body formation	Silencing compartiment	Dense *XIST* coating	H3K27me3 enrichment	H3K4me2 paucity	H3/H4 acteylation paucity	"Cancer-specific" escapees	Local heterochromatin formation
WI-38 / HMEC<br>Non tumoral cells		++	++	++	++	++	++	0%	++
ZR-75-1<br>Luminal breast cancer		+/-	+/-	+/-	+/-	+/-	+/-	9%<br>10/114	+/-
SK-BR-3<br>HER2+ breast cancer		+/-	+/-	+	+	+	+/-	8%<br>5/62	+/-
MDA-MB-436<br>Basal-like / BRCA1 null		+/-	+/-	+/-	+/-	+	+/-	14%<br>11/77	+/-
		-	?	-	-	+	+/-	13%<br>9/72	+/-

B

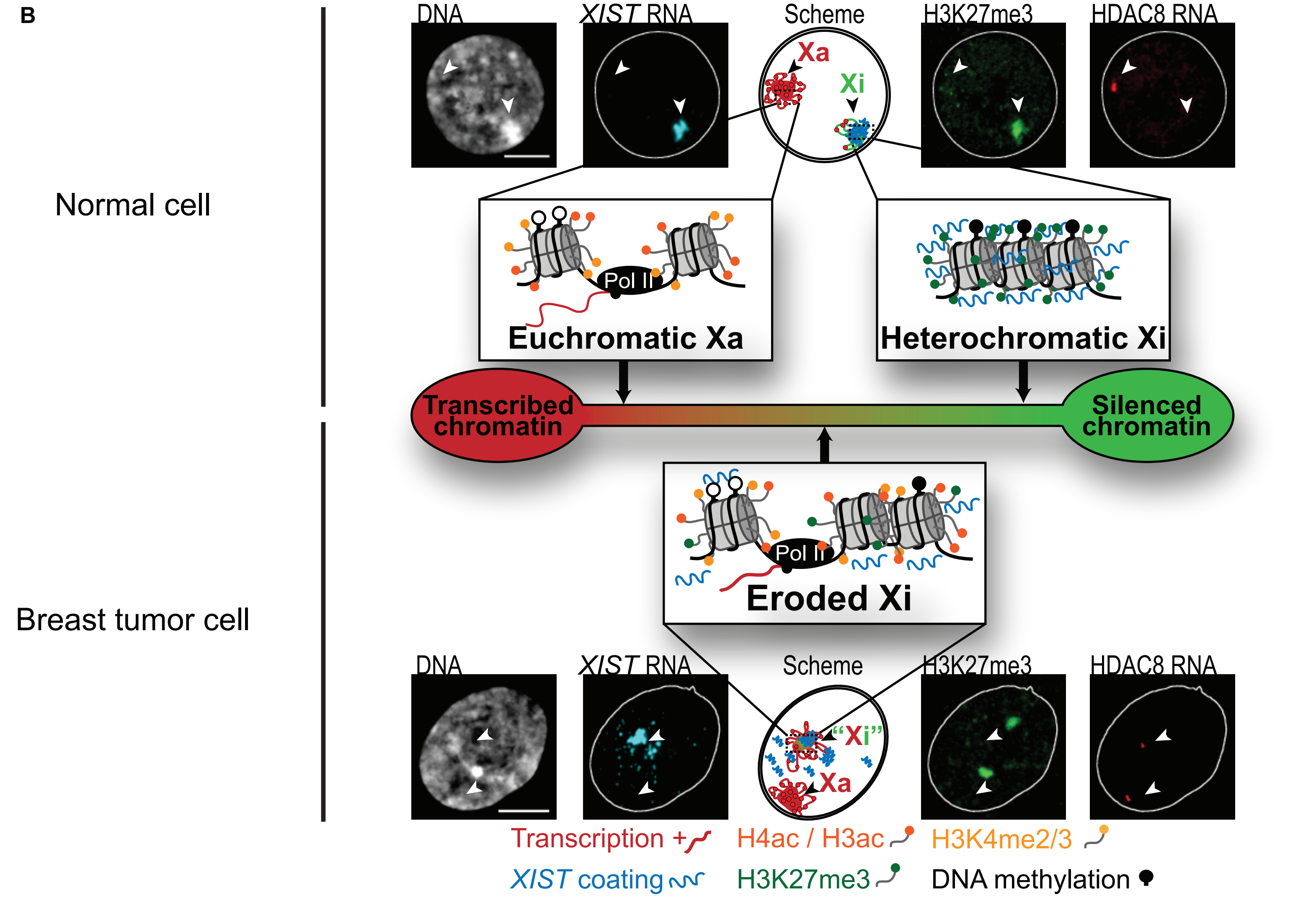

Figure S10, Chaligné et al
