# Supplementary material for: The inactive X chromosome is epigenetically unstable and transcriptionally labile in breast cancer
Source: Genome Res. 2015 Apr;25(4):488–503. doi: 10.1101/gr.185926.114 (PMC4381521; doi:10.1101/gr.185926.114)
Supplement: Supplemental Material [file supp_gr.185926.114_TableS3.docx]

| **Pyrosequencing primers** | |  |  |  |  |
| --- | --- | --- | --- | --- | --- |
| ***Gene*** | ***SNP*** |  |  | ***Primer*** |  |
|  |  |  |  |  |  |
| *NXT2* | rs3204027 | Fw | 5' | TGGAGTCAAAGCCATTGTTATCT | 3' |
|  |  | Rv | 5' | **Biot-**GGGGGATGTGTGATACATAAGAAA | 3' |
|  |  | Seq | 5' | GGAAGACTGGAAGACTTAA | 3' |
|  |  |  |  |  |  |
| *APOOL* | rs3747422 | Fw | 5' | GCGGAGAAAAGGCGGTAA | 3' |
|  |  | Rv | 5' | **Biot-**CAGAGAGGGACAAATCAGTTCG | 3' |
|  |  | Seq | 5' | CGGGGTGGGGCTGTC | 3' |
|  |  |  |  |  |  |
| *TBL1X* | rs16985675 | Fw | 5' | **Biot-**GTTTTCCCTAACAATTTGGACACT | 3' |
|  |  | Rv | 5' | CATCGGTACAAACTGGTCTTTGA | 3' |
|  |  | Seq | 5' | TCCTTTGTGAGAGCAAT | 3' |
|  |  |  |  |  |  |
| **Allele specific assay** |  |  |  |  |  |
| ***Gene*** | ***SNP*** |  |  | ***Applied biosystems assay reference*** |  |
|  |  |  |  |  |  |
| *NXT2* | rs3204027 |  |  | C_11174485_20 |  |
| *APOOL* | rs4828121 |  |  | C_2457746_20 |  |
| *HDAC8* | rs5912136 |  |  | C_29195024_20 |  |
| *HDAC8* | rs497551 |  |  | C_620029_10 |  |
| *CLCN4* | rs2240018 |  |  | C_2616133_1 |  |
|  |  |  |  |  |  |
|  |  |  |  |  |  |
| **Gene expression primers** | |  |  |  |  |
| ***Gene*** | ***RefSeq*** |  |  | ***Primer*** |  |
|  |  |  |  |  |  |
| *FAM46D* | NM_152630 | Fw | 5' | CAGGTGATGCAGAACTCTGGTAA | 3' |
|  |  | Rv | 5' | ACCAACCAGAAAAACGTGTCTTC | 3' |
| *MAGEA4* | NM_001011550 | Fw | 5' | CCAACCGGAGGACAGGATT | 3' |
|  |  | Rv | 5' | GGGACCCACAGGCAGATCTT | 3' |
| *MAGEA6 &MAGEA3* | NM_175868 & NM_005362 | Fw | 5' | GATCTGCCAGTGGGTCTCCAT | 3' |
|  |  | Rv | 5' | TGCTCAAGAGGCATGATGACTCT | 3' |
| *MAGEC2* | NM_016249 | Fw | 5' | GAAGAAACCCCGGCCTGTAC | 3' |
|  |  | Rv | 5' | TTCGTCACCACCTGGTTCCT | 3' |
| *TBL1X* | NM_001139467 | Fw | 5' | GCCTTGAATGGCAACTGATA | 3' |
|  |  | Rv | 5' | GATTTTCTTCCACCCTCACG | 3' |
| *TBP* | NM_001172085 | Fw | 5' | CAGGAGCCAAGAGTGAAGAACA | 3' |
|  |  | Rv | 5' | CCAGGAAATAACTCTGGCTCATAAC | 3' |
| *XIST* | NR_001564 | Fw | 5' | CTCGGACAGCTGTAAAGAAGAGTCT | 3' |
|  |  | Rv | 5' | AATGTCCAAGAGGAGCCTAAGGA | 3' |
|  |  |  |  |  |  |
|  |  |  |  |  |  |
|  |  |  |  |  |  |
| **Epityper® primers** |  |  |  |  |  |
| ***Gene*** | ***RefSeq*** |  |  | ***Primer*** |  |
|  |  |  |  |  |  |
| *HDHD1* | NM_001135565 | Fw | 5' | ATAGTAGTAATTTTGGAGTAGGTAGGTAAG | 3' |
|  |  | Rv | 5' | TTCTTCTAAATAAATAATATACAACC | 3' |
| *TBL1X* | NM_001139467 | Fw | 5' | GTTTTTAGGAGTTTGAATAATATTT | 3' |
|  |  | Rv | 5' | AACCCAAAAACAACAACAAATCTAC | 3' |
| *CLCN4* | NM_001830 | Fw | 5' | GAAAGGTTAGGTAAGTTGTATATATTAAG | 3' |
|  |  | Rv | 5' | AACCCCACTTTAAATTTCTAAAAAC | 3' |
| *RAB9A* | NM_004251 | Fw | 5' | GGGAGGATATTTTAGGAGTAAT | 3' |
|  |  | Rv | 5' | CCTCTCCCCAAAAATATAAAAAC | 3' |
| *TRAPPC2* | NM_001011658 | Fw | 5' | GAGGGGTGGAGGTATAGGATTAATA | 3' |
|  |  | Rv | 5' | CTACCCTCAAAACCCTACCTCTATC | 3' |
| *KDM5C* | NM_004187 | Fw | 5' | GGATTTTTGGTTTAATATTTTTTTT | 3' |
|  |  | Rv | 5' | AATCAACCTAAAACTCTCAAAACAC | 3' |
| *HUWE1* | NM_031407 | Fw | 5' | GTTATGTTTTTTTAGTAGTTTTTTT | 3' |
|  |  | Rv | 5' | CCCTATCATCCTCTATAATTACATTTACTA | 3' |
| *HDAC8* | NM_001166418 | Fw | 5' | TTTTTTATTTTTATTTGTTTATTGGA | 3' |
|  |  | Rv | 5' | CTAATCTAAAAAATCCCATCCATTC | 3' |
| *FAM46D* | NM_152630 | Fw | 5' | AAGAGTAGTTTTTTAAATAGGAAAGGATAT | 3' |
|  |  | Rv | 5' | AAAAACCCAAAATAAAAACTAAAAC | 3' |
| *HDX* | NM_144657 | Fw | 5' | AGGAGGATAGAGGTATTTGGTTTTT | 3' |
|  |  | Rv | 5' | CCCTCCCAACTCTTAAATTCTAAAC | 3' |
| *APOOL* | NM_198450 | Fw | 5' | GTTTAGGTTGAAGTGTAGTGG | 3' |
|  |  | Rv | 5' | ATCCAAATAAAAACTTAATACCCAAATATA | 3' |
| *SYTL4* | NM_001174068 | Fw | 5' | GTTTTGGAGAAAGAAAAGTGGT | 3' |
|  |  | Rv | 5' | AATCCCAAAACTCCCTTTTAAACTA | 3' |
| *NUP62CL* | NM_017681 | Fw | 5' | GGTGGTAGTTGATTAGGTGTTTTTT | 3' |
|  |  | Rv | 5' | CCAATAAAATAAATTTATAAAATTTTC | 3' |
| *NXT2* | NM_001242617 | Fw | 5' | TATGTTTATAAAAGTAAAGTGGTTTTTAAA | 3' |
|  |  | Rv | 5' | TACTAACCAAACTAAAAATAAAACC | 3' |
| *ACSL4* | NM_004458 | Fw | 5' | TAGATAGTTTTGGTTTAAGGAAAAA | 3' |
|  |  | Rv | 5' | ACTAACTCTACCACACCACC | 3' |
| *DOCK11* | NM_144658 | Fw | 5' | GGGGTGTGAGTTAAAGAGTTGATT | 3' |
|  |  | Rv | 5' | CAAATCCTCCATCCCTAAACTAAC | 3' |
| *Il13RA1* | NM_001560 | Fw | 5' | GGGGATTGTTAAGGTTTTAGTT | 3' |
|  |  | Rv | 5' | ACCTCCTAAACTCTTCCCACTACA | 3' |
| *AIFM1* | NM_001130846 | Fw | 5' | GGTTGTTTGGAATGGGTTAGTTAT | 3' |
|  |  | Rv | 5' | ATCCCTCCTTAAACAAATTCCTTAA | 3' |
| *MAGEC2_#1* | NM_016249 | Fw | 5' | TTTTTTGGGGTTATTTTTTTGTTT | 3' |
|  |  | Rv | 5' | AACTACACCAAACCACTCCCTACTA | 3' |
| *MAGEC2_#2* | NM_016249 | Fw | 5' | TTTTTTGGTTGTTGGATTTTTTATT | 3' |
|  |  | Rv | 5' | AAAACCACTCACACTATCACTCTCTT | 3' |
| *ZNF275* | NM_001080485 | Fw | 5' | TATTTGAGGGTTTATAGTAGAGAGAAGTTT | 3' |
|  |  | Rv | 5' | TACTTAATAAAATCAAAAAACCCAC | 3' |
| *VBP1* | NM_003372 | Fw | 5' | GTGGGGTTAGGTTAGGTTTTTATTT | 3' |
|  |  | Rv | 5' | ACTTAACTCAACTCCTTCCAACAAT | 3' |
| Tag, 5' on Fw primer |  |  |  | AGGAAGAGAG |  |
| T7, 5' on Rv primer |  |  |  | CAGTAATACGACTCACTATAGGGAGAAGGCT |  |
|  |  |  |  |  |  |
